# Dissecting the Genome-Wide Evolution and Function of R2R3-MYB Transcription Factor Family in *Rosa chinensis*

**DOI:** 10.3390/genes10100823

**Published:** 2019-10-18

**Authors:** Yu Han, Jiayao Yu, Tao Zhao, Tangren Cheng, Jia Wang, Weiru Yang, Huitang Pan, Qixiang Zhang

**Affiliations:** 1 Beijing Key Laboratory of Ornamental Plants Germplasm Innovation & Molecular Breeding, National Engineering Research Center for Floriculture, Beijing Laboratory of Urban and Rural Ecological Environment, Key Laboratory of Genetics and Breeding in Forest Trees and Ornamental Plants of Ministry of Education, School of Landscape Architecture, Beijing Forestry University, Beijing 100083, China; 15201425912@126.com (Y.H.); jiayaoyubjfu@126.com (J.Y.); chengtangren@163.com (T.C.); 13910229248@163.com (J.W.); yweiru@163.com (W.Y.); 13601231063@163.com (H.P.); 2 VIB-UGent Center for Plant Systems Biology, Technologiepark, Zwijnaarde 71, 9052 Ghent, Belgium; 3 Beijing Advanced Innovation Center for Tree Breeding by Molecular Design, Beijing Forestry University, Beijing 100083, China

**Keywords:** R2R3-MYB, gene family evolution, synteny network, phenylpropanoid pathway

## Abstract

*Rosa chinensis*, an important ancestor species of *Rosa hybrida*, the most popular ornamental plant species worldwide, produces flowers with diverse colors and fragrances. The R2R3-MYB transcription factor family controls a wide variety of plant-specific metabolic processes, especially phenylpropanoid metabolism. Despite their importance for the ornamental value of flowers, the evolution of *R2R3-MYB* genes in plants has not been comprehensively characterized. In this study, 121 predicted *R2R3-MYB* gene sequences were identified in the rose genome. Additionally, a phylogenomic synteny network (synnet) was applied for the *R2R3-MYB* gene families in 35 complete plant genomes. We also analyzed the *R2R3-MYB* genes regarding their genomic locations, Ka/Ks ratio, encoded conserved motifs, and spatiotemporal expression. Our results indicated that *R2R3-MYBs* have multiple synteny clusters. The *RcMYB114a* gene was included in the Rosaceae-specific Cluster 54, with independent evolutionary patterns. On the basis of these results and an analysis of *RcMYB114a*-overexpressing tobacco leaf samples, we predicted that *RcMYB114a* functions in the phenylpropanoid pathway. We clarified the relationship between *R2R3-MYB* gene evolution and function from a new perspective. Our study data may be relevant for elucidating the regulation of floral metabolism in roses at the transcript level.

## 1. Introduction

The v-myb myeloblastosis viral oncogene homolog (MYB) proteins form one of the largest transcription factor families, widely distributed in all eukaryotic organisms [1]. The MYB proteins are identified based on their highly conserved N-terminal DNA-binding domain [-W-(X_19_)-W-(X_19_)-W-…-F/I-(X_18_)-W-(X_18_)-W-], which can form three α-helices, with the second and third helices forming a helix-turn-helix motif [2,3], as well as a highly variable C-terminal activation or repression domain [4]. On the basis of the number of repeating MYB domains, the MYB family has been divided into the following four subfamilies: 1R-MYB (or MYB-related), R2R3-MYB (2R-MYB), R1R2R3-MYB (3R-MYB), and 4R-MYB [2,5]. The R2R3-MYB subfamily is the largest, with members containing two MYB domain repeats that are similar to the R2 and R3 repeats of their vertebrate homologs, c-MYB [6]. Many R2R3-MYB proteins influence processes related to plant growth and development, such as cell-cycle regulation, meristem formation, and cellular morphogenesis [7]. The R2R3-MYBs are also vital for various plant responses, including biotic and abiotic stresses, phytohormones, and diverse environmental signals [8,9,10,11,12].

One of the most important roles of R2R3-MYBs involves the regulation of the synthesis of phenylpropanoid-derived compounds, which represent a diverse family of secondary metabolites [13]. In 1987, the first isolated MYB gene, *ZmC1* from *Zea mays*, was revealed to affect anthocyanin biosynthesis [14]. Since then, many MYB genes have been identified and characterized in various plant species regarding their sequence and potential functions [2,15]. The release of genome data has enabled phylogenetic analyses of the MYB family at the genome level in several plant species, including *Arabidopsis thaliana* [16], *Z. mays* [17], *Glycine max* [18], *Malus domestica* [19], and *Eucalyptus grandis* [20]. The conserved subgroup in *A. thaliana* can be used to predict the functions of *R2R3-MYB* genes in nonmodel species, because genes in the same subgroup are thought to have similar roles [21]. In addition to phylogenetic and conserved subgroup analyses to functionally characterize *R2R3-MYB* genes, investigations of whole-genome synteny have also been conducted [22]. An analysis of synteny may clarify the evolution of gene families by revealing local conserved gene arrangements in a genome. Moreover, a comparative analysis of synteny between species may be relevant for elucidating the evolutionary trajectory of genes and genomes as well as the final traits of organisms [23,24]. Building a network of all of the syntenic relationships among *R2R3-MYB* genes across many species can provide new insights into the *R2R3-MYB* gene evolutionary history.

Rose species are the most commonly cultivated ornamental plants worldwide. There are currently more than 35,000 rose cultivars [25]. The considerable ornamental value of rose plants is due to their diverse floral colors, pleasant floral fragrance, and recurrent flowering trait. *Rosa chinensis* ‘Old Blush’ has made important contributions to the modern rose cultivars (*Rosa hybrida*), and a high-quality genome sequence for ‘Old Blush’ has been obtained [26]. In roses, the main compounds responsible for flower colors (i.e., anthocyanidins and flavonoids) and volatile compounds (i.e., 3, 5-dimethoxytoluene and phenylethyl alcohol) are synthesized by the phenylpropanoid pathway [27,28,29]. Therefore, R2R3-MYB proteins are likely to be involved in the regulation of rose flower colors and floral development, but more evidence is needed to confirm their roles.

In this study, we applied a phylogenomic synteny network (synnet) of *R2R3-MYB* genes across 35 plant genomes to identify the rose *R2R3-MYB* genes and investigate their origins and evolutionary positions. We also analyzed the *R2R3-MYB* genes based on their genomic locations, phylogenetic relationships, and encoded conserved motifs. Moreover, the R2R3-MYB transcript abundance patterns in rose petals and other tissues were assessed by RNA sequencing (RNA-seq). On the basis of these analyses, we may have identified several R2R3-MYBs involved in the regulation of the metabolism of developing rose flowers. Furthermore, the data presented herein may be useful for future studies that examine the regulation of rose floral metabolism at the transcript level.

## 2. Materials and Methods

### 2.1. Plant Growth and Sample Collection

*Rosa chinensis* ‘Old Blush’ and *Nicotiana benthamiana* plants were grown in a glasshouse at Beijing Forestry University (Beijing, China) under a 12 h (25 °C) day/12 h (18 °C) night cycle. The samples were collected from 3-year-old rose plants and immediately placed in a 5 mL centrifuge tube. Specifically, the petals were harvested during the following four typical developmental stages: FB_GP (green petals in the flower bud), FB_CP (petals changing colors in the flower bud), FB_PP (pink petals in the flower bud), and OF_PP (pink petals on the open flower). The root, stem, leaf, prickle, stamen, pistil, and ovary were also harvested. For each collected plant material, samples from six plants were combined to form one sample and then frozen in liquid nitrogen.

### 2.2. Plant Genome Analysis and Pairwise Whole-Genome Comparisons

The genomes of 35 plant species were included in the analyses (Supplemental Table S1). The species status was checked with the R package plant list. The phylogenetic relationships were analyzed with Phylomatic (version 3), and a phylogenetic tree was constructed with the Figtree program (version 1.4.4). All-against-all pairwise comparisons of whole-genome sequences were completed with a GitHub script (https://github.com/zhaotao1987/SynNet-Pipeline) to detect all synteny blocks.

### 2.3. Identification of R2R3-MYB Gene Models

The Pfam annotations based on the conserved protein domain PF00249 were used for identifying the *R2R3-MYB* genes [30]. Hidden Markov Models (HMMs) retrieved from the Pfam 3.0 database were used to search 35 plant genomes with the hmmscan program of the HMMER3.0 package [31] to identify R2R3-MYB proteins with two DNA-binding domains (PF00249). All R2R3-MYB proteins with significant hits (*e*-value < 0.001) that are encoded by the genomes of 35 species (nodes) are listed in Supplemental Table S2.

### 2.4. Calculation of Syntenic Blocks and Construction of a Synteny Network for R2R3-MYBs

The Synets method was used for calculating syntenic blocks, constructing networks, and detecting clusters [32]. The default parameters of the MCScanX program [33] (minimum match size for a collinear block = 5 genes, maximum gaps allowed = 25 genes) were used to compute genomic collinearity. Information regarding all syntenic blocks (edges, with headers Locus_1 and Locus_2) is provided in Supplemental Table S3. The syntenic blocks containing all of the *R2R3-MYB* genes in the 35 analyzed species were used to build the synnet, visualized with Cytoscape (version 3.6.1) [34] and Gephi 0.9.1 [35].

### 2.5. Network Clustering

The CFinder program was used to implement the clique percolation method [36,37] and locate clique communities (*k* = 3) for the R2R3-MYB synnet to identify clusters of gene nodes (Supplemental Tables S4 and S5). The clusters were visualized with Gephi 0.9.1 [35]. For each genome, all clusters were decomposed to the number of involved syntenic genes (Supplemental Table S6). The dissimilarity index of all clusters was calculated according to the Jaccard method of the vegan package [38] and then hierarchically clustered and visualized with ward.D and pheatmap, respectively.

### 2.6. Chromosomal Localization and Phylogenetic Analysis

Chromosomal location information was obtained from the gff3 file of the *R. chinensis* genome database (RchiOBHm-V2) (Supplemental Table S9). The Mapchart software [39] was used to visualize the chromosomal locations of all rose *R2R3-MYB* genes.

The amino acid sequences of 121 RcMYB proteins (*R. chinensis*) and 136 AtMYB proteins (*A. thaliana*) were used to analyze the phylogenetic relationships between the R2R3-MYBs of these two plant species (Supplemental Table S10). Multiple sequences were aligned with the ClustalX program (version 2.0), and a phylogenetic tree was constructed according to the neighbor-joining method of MEGA 6.0, with 1000 bootstrap replicates [40,41]. The iTOL and evolview online tools were used to annotate the phylogenetic tree [42]. Conserved motifs were detected based on the multiple sequence alignment by ClustalX (version 2.0). All sequences were stored in a fasta file. WebLogo was used to generate sequence logos.

### 2.7. Calculation of Ka/Ks Ratios of Gene Pairs

To determine whether *R2R3-MYB* genes evolved under positive selection, the *A. thaliana* and *R. chinensis R2R3-MYB* genes were aligned with MEGA 6.0. The DnaSP (version 6.0) program was then used to calculate the synonymous substitution (Ks) and nonsynonymous substitution (Ka) rates. The Ka/Ks ratio indicates the selection pressure on genes as follows: Ka/Ks > 1 (positive selection), Ka/Ks < 1 (negative selection), and Ka/Ks = 1 (neutral selection) (Supplemental Table S12) [43].

### 2.8. Transcriptome Sequencing, Functional Annotation, and Analysis of Differentially Expressed Genes

Total RNA was extracted from the various collected plant materials (root, stem, leaf, prickle, stamen, pistil, and ovary) with the SV Total RNA Isolation Kit (Promega, Madison, WI, USA), and the RNA purity was checked using the NanoPhotometer^®^ spectrophotometer (Implen, West Lake Village, CA, USA). The Qubit® RNA Assay Kit in the Qubit® 2.0 Flurometer (Life Technologies, Carlsbad, CA, USA) was used to measure the RNA concentration, and the RNA Nano 6000 Assay Kit of the Bioanalyzer 2100 system (Agilent Technologies, Palo Alto, CA, USA) was used to assess the RNA integrity. A total amount of 1 µg RNA per sample was used as the input material for the RNA sample preparations. The RNA samples were used as templates to construct libraries; each kind of sample had two biological replications and a total of 12 libraries. A NEBNext^®^ Ultra^TM^ RNA Library Prep Kit (New England Biolabs, MA, USA) was used to generate the libraries, following the manufacturer’s recommendations, and index codes were added to attribute sequences to each sample. The library fragments were purified using the Agencourt AMPure XP system (Beckman Coulter, Brea, CA, USA) and 250–300 bp cDNA fragments were selected. The Agilent Bioanalyzer 2100 system (Agilent Technologies) were used to assess the library quality. The libraries were sequenced using an Illumina HiSeq™ 4000 system (Illumina, San Diego, CA, USA) at the Novogene Bioinformatics Institute (Beijing, China). Each library was sequenced to at least 6 Gb clean bases.

All raw data were submitted as a BioProject (PRJNA546486) to the NCBI (National Center for Biotechnology Information) Sequence Read Archive (accession number SUB5725876). Clean reads were obtained by removing reads containing adapter sequences, reads containing ploy-N, and low quality reads from raw data and were calculated by their Q20, Q30, GC contents, and error rates. All reads were quantified with StringTie [44], and the information is shown in Supplemental Table S14. All the downstream analyses were based on the clean reads with high quality. The *R. chinensis* ‘Old Blush’ reference genome and gene model annotation files were downloaded from an online genome portal (RchiOBHm-V2) [26]. HISAT (version 2.0.4) was used for building the index of the reference genome [45] and aligning the paired-end clean reads to the reference genome. HTSeq v0.9.1 was used to count the read numbers mapped to each gene (Supplemental Table S15) [46]. The expected number of fragments per kilobase of transcript sequence per million base pairs sequenced (FPKM) of each gene was calculated based on the length of the gene and the read count mapped to this gene [47]. Differential expression analysis of two groups (two biological replicates per group) was performed using the DESeq R package (1.18.0) [48]. The resulting *p*-values were adjusted using the Benjamini and Hochberg’s approach for controlling the false discovery rate. Genes with an adjusted *p*-value < 0.05, found by DESeq, were assigned as differentially expressed. All read annotations were retrieved from the RchiOBHm-V2 ‘Old Blush’ genome annotation database, including gene descriptions and Blast2GO annotations.

Except for the new transcriptome sequencing of other rose tissues, we also analyzed the published sequencing data of rose petals under the same data treatments. The FPKM method [47] was used to normalize the differential expression of *RcMYB* genes in petals at different developmental stages (BioProject PRJNA351281; accession number SRP092271, Supplemental Table S13) [49] and in other rose tissues (Supplemental Table S16). Heat maps representing gene expression levels were drawn with the R package pheatmap.

### 2.9. Validation of Gene Expression by a qRT-PCR Assay

The gene expression levels in the following rose tissues were validated by a quantitative real-time polymerase chain reaction (qRT-PCR) assay: root, stem, leaf, prickle, stamen, pistil, ovary, and petals collected at four developmental stages (FB_GP, FB_CP, FB_PP, and OF_PP). The total RNA was extracted with the SV Total RNA Isolation Kit (Promega). First-strand cDNA was synthesized from 1.0 µg total RNA with the PrimeScript RT Reagent Kit with gDNA Eraser (Takara Bio, Shiga, Japan). The qRT-PCR assay was completed with the CFX96™ Real-Time PCR system (Bio-Rad Laboratories, Hercules, CA, USA) and a reaction solution comprising 10 µL SYBR Premix Ex Taq (Takara Bio), 0.4 µL 10 µM forward and reverse transcript-specific primers (Supplemental Table S17), 2 µL cDNA, and 7.2 µL sterile distilled water. The PCR program was as follows: 95 °C for 30 s, 40 cycles of 95 °C for 5 s and 60 °C for 30 s, and a final melting curve analysis stage of 95 °C for 15 s, 60 °C for 1 min, and 95 °C for 15 s. The relative gene expression levels were calculated with the 2^−ΔΔCq^ method (Young et al., 2010) and were normalized against the expression level of the endogenous reference gene (*RcActin*) [49]. Each sample was examined with three biological replicates, each consisting of three technical replicates. The Origin9 program (OriginLab, Northampton, MA, USA) was used to generate histograms.

### 2.10. Transient Overexpression of RcMYB114a in Tobacco

To generate the *RcMYB114a* overexpression construct, a full-length *RcMYB114a* cDNA sequence was obtained by a PCR amplification of rose petal cDNA with the OE-*RcMYB114a* forward and reverse primers (Supplemental Table S17) under the following conditions: 94 °C for 2 min; 35 cycles of 98 °C for 10 s, 54 °C for 30 s, and 68 °C for 1 min; and 68 °C for an additional 2 min (KOD-plus, Toyobo, Japan). The purified PCR product was ligated to the pDONR221 entry vector and sequenced, after which it was incorporated into the pH7WG2D expression vector. The pH7WG2D vector and the pH7WG2D-*RcMYB114a* recombinant plasmid were inserted into *Agrobacterium tumefaciens* EHA105 cells, which were then incubated at 28 °C in Luria-Bertani liquid medium containing 10 mM MES [2-(4-morpholino)ethanesulfonic acid] and 20 μM acetosyringone. The transformed *A. tumefaciens* cells were harvested when the optical density at 600 nm (OD_600_) of the culture reached 0.2. The cells were resuspended in infection buffer (10 mM MgCl_2_, 10 mM MES, pH 5.6, and 150 μM acetosyringone) for a final OD_600_ of 0.4. The resuspended cells were maintained at room temperature for 2 h. The suspension with cells carrying pH7WG2D-*RcMYB114a* was gently injected into the epidermis on the right side of a tobacco leaf with a sterile needle-free 1 mL syringe. The control suspension (i.e., with cells carrying the empty pH7WG2D vector) was injected into the epidermis on the left side of the same leaf. The leaf inoculation was completed with three biological replicates, each consisting of 10 leaves. After incubating the infected leaves for 40–48 hours under the plant growth conditions, the left and right sides of each leaf were collected separately for a qRT-PCR assay, which was completed as described in the previous section.

## 3. Results

### 3.1. Identification of R2R3-MYB Genes in 35 Genomes

**Figure 1:**
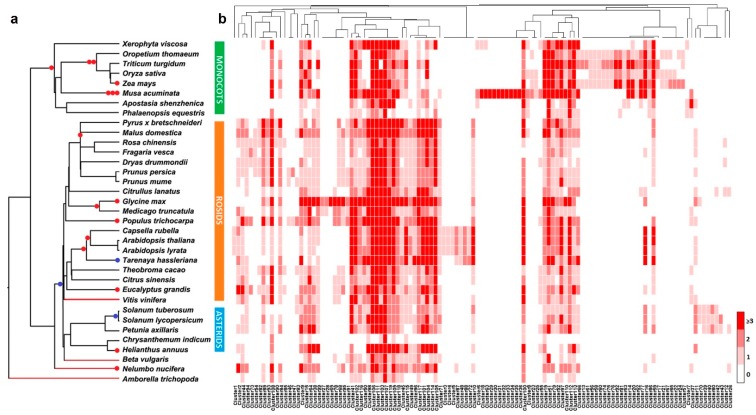
Phylogenetic relationships among 35 species and the distribution of syntenic *R2R3-MYB* genes in these species. (**a**) Phylogenetic tree comprising 35 species. Red branches indicate the basal rosid *Vitis vinifera*, the basal eudicots *Beta vulgaris* and *Nelumbo nucifera*, and the basal angiosperm *Amborella trichopoda*. Red and blue dots indicate whole-genome duplication and whole-genome triplication events, respectively. (**b**) Phylogenetic profile revealing the number and distribution of *R2R3-MYB* genes in 119 clusters (*k* = 3) for 35 species (Supplemental Table S6). Rows are arranged according to the species listed in panel (**a**). Each cell indicates the number of *R2R3-MYB* genes in one species.

We constructed a phylogenetic tree based on 35 plant species with published genome data, including 8 monocots, 19 rosids, 5 asterids, *Beta vulgaris* and *Nelumbo nucifera* (eudicots that are not rosids or asterids), and an early diverging angiosperm (*Amborella trichopoda*) (Supplemental Table S1). In Figure 1a, the whole-genome duplication (WGD) and whole-genome triplication (WGT) events are indicated with red and blue dots, respectively. *Rosa chinensis* was most closely related to *Fragaria vesca* among the included species. We performed a genome-wide sequence homology search to identify the R2R3-MYBs in the 35 species. Eight R2R3-MYB proteins (AT2G36890, AT2G47190, AT3G49690, AT3G60460, AT4G38620, AT5G23000, AT5G35550, and AT5G56110) were used to build profile HMMs. On the basis of these profiles, we identified 4648 R2R3-MYBs in 35 plant species, with considerable differences in the copy numbers among the examined genomes (Supplemental Table S2).

Synteny networks have been used to identify sequence homologies and elucidate the evolutionary history of genes in plants [32,50]. The synnet applied in the current study consisted of nodes representing *R2R3-MYB* genes, with edges that indicated synteny and sequence similarity (Figure S1). We detected 58,500 edges (pairwise syntenic connections between genes) and 4,648 nodes (genes connected to other genes) (Supplemental Table S2 and S3). Communities (node clusters) can be detected in the synnet with a published community detection method [50]. A total of 119 clusters were obtained with a k-clique percolation clustering method (*k* = 3) implemented by CFinder and were visualized with Gephi [35] (Supplemental Tables S4 and S5). These clusters were used to generate a phylogenetic profile. Detailed edgelist information for each cluster is provided in Supplemental Table S5. The 119 clusters in the phylogenetic profile for the *R2R3-MYB* genes in 35 species are highlighted in Figure 1b and Supplemental Table S6. These clusters were also investigated to clarify the syntenic relationships among the *R2R3-MYB* genes. The clusters were subdivided into the following six evolutionary categories based on the gene specificity in each cluster: angiosperm-wide (25 clusters), eudicot-specific (35 clusters), monocot-specific (28 clusters), rosid-specific (18 clusters), asterid-specific (4 clusters), and species-specific (9 clusters) (Supplemental Table S7). Moreover, 25 clusters comprised R2R3-MYBs from all 35 plant species, including *A. trichopoda*, monocots, and eudicots, suggesting that these MYBs are highly conserved and originate in angiosperms. The specificity of each cluster is indicated in Figure 1b.

### 3.2. Some Lineage-Specific Clusters of R2R3-MYBs

**Figure 2:**
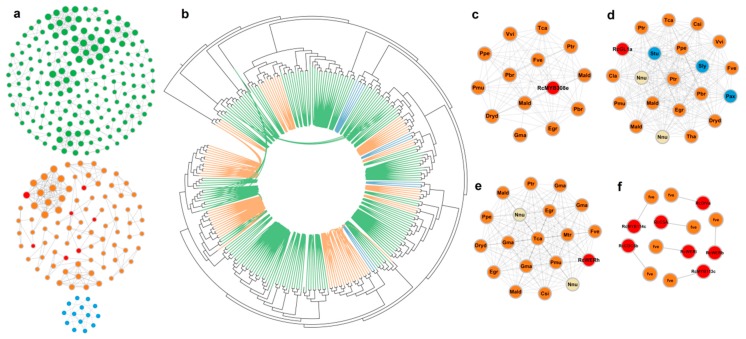
Partial clustering analysis. (**a**) Lineage-specific clusters of monocots (green nodes, top), rosids (orange nodes, middle), and asterids (blue nodes, bottom). The *Rosa chinensis* R2R3-MYBs are marked in red. (**b**) A neighbor-joining phylogenetic tree (Poisson correction) of all lineage-specific clusters. Connected branches indicate synteny between the gene pairs. The green, orange, and blue lines indicate genes belonging to monocots, rosids, and asterids, respectively. (**c**–**e**) Clusters of Clusters 93, 94, and 95. Species abbreviations for each MYB are provided in the middle of each node. Details regarding the species abbreviations are listed in Supplemental Table S1. The *Beta vulgaris*, *Nelumbo nucifera,* and *Amborella trichopoda* nodes are in beige. The rose nodes are in red, with gene names provided. (**f**) Seven independent syntenic *R2R3-MYB* gene pairs between *R. chinensis* and *Fragaria vesca* are presented. The rose nodes are in red, with gene names provided.

To further clarify the evolutionary events affecting the R2R3-MYB homologs in various plant species, we developed clusters for monocots, rosids, and asterids (Figure 2a and Supplemental Table S7). We also determined the pairwise syntenic relationships among the *R2R3-MYB* genes of all lineage-specific clusters of monocots, rosids, and asterids in the phylogenetic tree (Figure 2b). Several genes from monocot or rosid species in distal gene clades were syntenically connected. The phylogenetic and network-based analyses clearly indicated that genes from lineage-specific clusters were connected. An analysis of the identified *R2R3-MYB* genes, presented in Figure 2a, revealed that the monocot-specific clusters contained the most syntenic homologs (syntelogs), implying these genes share a common monocot origin. In contrast, the asterid-specific clusters had the fewest syntelogs. The lineage-specific rosid clusters included seven *R. chinensis MYB* genes (Figure 2a, red dots), which may have been the result of rosid-independent evolutionary events.

The R2R3-MYB syntelogs can be detected with the Synets method [32,50]. The homologs on the corresponding chromosomes of different species as well as the paralogs in one species were clearly presented in the R2R3-MYB clusters. We developed an additional three clusters (Figure 2c–e). Cluster 93 contained 14 genes from 12 species (i.e., peach, grape, cacao, *Prunus mume*, pear, strawberry, poplar, *Dryas drummondii*, apple, soybean, eucalyptus, and rose). This cluster was rosid-specific and the apple and pear paralogs may have been the result of a WGD event (Figure 2c). Other clusters also appeared with similar regularities, such as Cluster 94 and Cluster 95 (Figure 2d,e). These results may be useful for identifying the *R. chinensis* R2R3-MYB orthologs and paralogs and for characterizing the specific evolutionary events that affected R2R3-MYBs. There were seven independent syntenic gene pairs between *R. chinensis* and *F. vesca* when *k* < 3, which was consistent with the relatively close evolutionary relationship between rose and strawberry (Figure 2f and Supplemental Table S8).

### 3.3. Physical Distribution of R2R3-MYB Genes on R. chinensis Chromosomes

**Figure 3:**
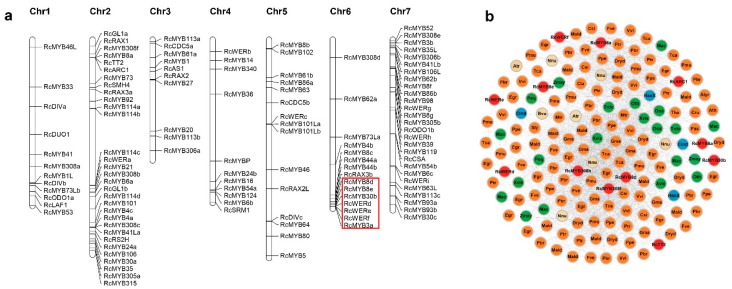
Physical positions of the 121 *R2R3-MYB* genes on seven *R. chinensis* chromosome scaffolds, and the synteny network of Cluster 108. (**a**) The physical distribution of *R2R3-MYB* genes on rose chromosomes. The tandemly distributed *MYB* genes on Chr6 are marked by a red frame. (**b**) Cluster 108 synteny network. Node colors represent the following: rosids (orange), monocots (green), asterids (blue), *R. chinensis* (red), and other species (beige). Gene names and species abbreviations are provided in the nodes of rose and other species, respectively.

We identified 121 *R. chinensis* R2R3-MYB proteins, which were named according to the annotations based on genome data (Supplemental Table S9). The genes having identical names but differentiated by a, b, c, and d refer to MYB transcription factors with the same annotations and positioned on closely linked loci. The physical locations of the genes encoding these 121 *MYBs* on seven *R. chinensis* chromosomes (Chr1–Chr7) were visualized with Mapchart (Figure 3a and Supplemental Table S9). As presented in Figure 3a, Chr2 had the most *RcMYB* genes (31, 25.6%), followed by Chr7 (27, 22.3%). In contrast, Chr3 only had 11 *R2R3-MYB* genes. Additionally, Ch6 contained seven tandemly distributed *RcMYB* genes (namely *RcMYB8d*, *RcMYB8e*, *RcMYB30b*, *RcWERd*, *RcWERe*, *RcWERf*, and *RcMYB3a*), all of which belonged to Cluster 108. An analysis of Cluster 108 revealed that it was an angiosperm-wide cluster and the tandem distribution of genes mostly occurred in Rosaceae species (Figure 3b and Supplemental Table S4).

### 3.4. Predicted Functions and Analysis of the Spatiotemporal Expression of R2R3-MYB Genes in R. chinensis

**Figure 4:**
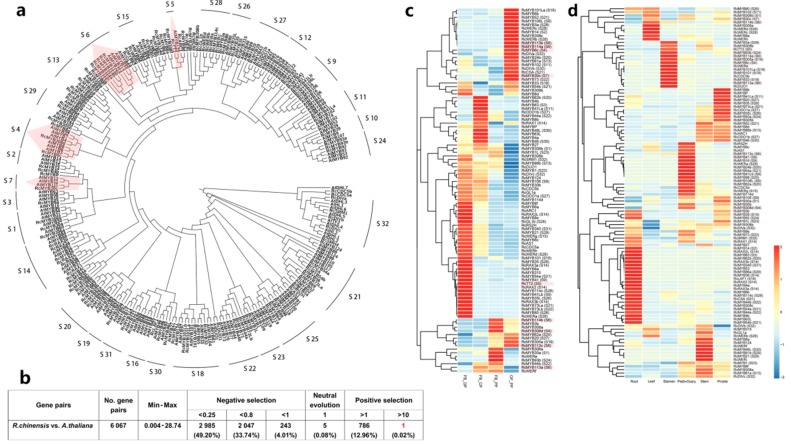
Phylogenetic relationships and *R2R3-MYB* gene expression heat maps. (**a**) Neighbor-joining phylogenetic tree constructed based on 257 predicted amino acid sequences (136 *Arabidopsis thaliana* proteins and 121 *R. chinensis* proteins). All subgroups had bootstrap values exceeding 50. The subgroup ID numbers are provided in the outer circle of the phylogenetic tree. Subgroups 4–7 are in pink. (**b**) Analysis of the Ka/Ks ratios for the *R. chinensis* and *A. thaliana R2R3-MYB* genes. (**c**) Analysis of *R2R3-MYB* genes differentially expressed during the following four petal developmental stages of ‘Old Blush’: FB_GP, green petals in the flower bud; FB_CP, petals changing colors in the flower bud; FB_PP, pink petals in the flower bud; OF_PP, pink petals on the open flower. The genes in Subgroups 4–7 are in pink. (**d**) Analysis of *R2R3-MYB* genes differentially expressed in other tissues of ‘Old Blush’ plants (i.e., root, leaf, stamen, pistil, ovary, stem, and prickle). The genes in Subgroups 4–7 are in pink.

We predicted the functions of rose R2R3-MYBs based on the conserved subgroups in *A. thaliana*. Additionally, an alignment of the *R. chinensis* and *A. thaliana* MYB protein sequences was used to construct a neighbor-joining phylogenetic tree (Figure 4a). Considering the topology of the tree and the clade support values (at least 50% bootstrap support), we subdivided the phylogenetic tree into 32 subgroups. Moreover, the information derived from 67 related articles was applied to predict gene functions. All of the relevant information is provided in Supplemental Table S11. Among all subgroups, S28 was specific to *R. chinensis* and S12 was specific to *A. thaliana*. However, 54 genes (40 *RcMYB* genes and 14 *AtMYB* genes) did not fit well into any subgroup (Supplemental Table S11).

We also calculated the Ks and Ka values for the *R. chinensis* and *A. thaliana R2R3-MYB* genes (Figure 4b and Supplemental Table S12). We identified 6067 homologous gene pairs. Overall, 86.95% (5275) of the homologous gene pairs had a Ka/Ks ratio less than 1, whereas 12.98% (787) of the homologous gene pairs had a Ka/Ks ratio greater than 1 (Figure 4b). One gene pair (*AtDHL5* and *RcMYB114b*) had a Ka/Ks ratio of 28.75. The *RcMYB114b* gene may have evolved under strong positive selection. However, most of the Ka/Ks ratios for the homologous genes were less than 0.8.

The R2R3-MYB mRNA levels also affected their functions. To examine the spatiotemporal *RcMYB* expression dynamics, the RNA-seq data for four typical petal developmental stages and six tissues in ‘Old Blush’ were analyzed (Figure S2 and Supplemental Tables S13–S16). Heat maps were used to visualize the tissue-specific, *RcMYB* expression patterns based on FPKM data (Figure 4c,d). Transcripts were detected for 100 *RcMYB* genes in the petals collected during four developmental stages. The *RcMYB* genes were more highly expressed in the FB_GP and OF_PP stages than in the other two stages. Additionally, 110 *RcMYB* genes were differentially expressed in various rose tissues, including the root, stem, leaf, stamen, prickle, pistil, and ovary. The *RcMYB* genes that were expressed at relatively high levels in specific tissues are presented in Figure 4d. Many *R2R3-MYB* genes were actively expressed in roses, especially in the petals.

We focused on the subgroups involved in the phenylpropanoid pathway (Subgroups 4–7 (S4–S7)). The *AtMYB* genes in these subgroups have been confirmed to influence anthocyanin biosynthesis, flavonol biosynthesis, and other pathways related to the development of flower colors and fragrances (references in Supplemental Table S11). Only *RcTT2* from S5 was highly expressed in the FB_GP stage. The genes encoding other MYBs in S4, S6, and S7 were highly expressed in the FB_PP and OF_PP stages, which correspond to the stages during which the flowers are opening (Figure 4c). Further analyses of the *RcMYB* expression levels in the other examined tissues revealed that *RcMYB* genes in S4–S7 were mainly expressed in the leaf, stamen, pistil, and ovary.

**Figure 5:**
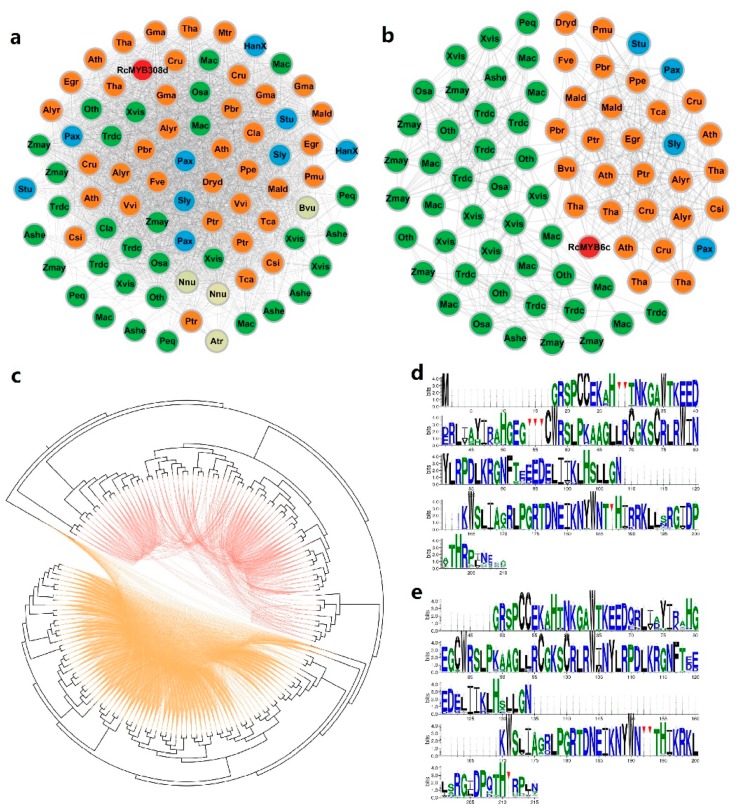
Comparative analysis of Clusters 112 and 59 in Subgroup 4. (**a**,**b**) Synteny networks of Clusters 112 and 59. Node colors represent the following: rosids (orange), monocots (green), asterids (blue), *R. chinensis* (red), and other species (beige). Gene names and species abbreviations are provided in the nodes of rose and other species, respectively. (**c**) Neighbor-joining phylogenetic tree of genes in Clusters 112 and 59. Connected branches indicate synteny between the gene pairs. Orange and red connections represent the synteny of Clusters 112 and 59, respectively. (**d**,**e**) Comparison of the myb-binding domain of all R2R3-MYBs in Cluster 112 (**d**) or Cluster 59 (**e**) based on sequence logos. Bits represent the conservation of an amino acid at a particular position. The positions with different patterns in Clusters 112 and 59 are indicated by red triangles. The size of each amino acid letter corresponds to how conserved the amino acid is.

Subgroup 4 included six *AtMYB* genes, namely *AtMYB3* (homolog of *RcMYB6c*), *AtMYB6*, *AtMYB8* belonging to Cluster 59, *AtMYB4* (homolog of *RcMYB308d*), *AtMYB7*, and *AtMYB32* belonging to Cluster 112 (Figure 4a and Supplemental Table S11). Although all S4 genes contribute to the phenylpropanoid pathway, they were divided into two clusters. All nodes were closely connected to each other in Cluster 112, but the edges were separated into two parts in Cluster 59 (Figure 5a,b). We constructed a phylogenetic tree for the two clusters to represent the syntenic connections among these genes (Figure 5c). The alignments of the 82 and 74 MYB proteins encoded by genes in Clusters 112 and 59 were built by WebLogo (Figure 5d,e). The differences of residues have been marked by red triangles in Figure 5d,e. Moreover, *RcMYB30c* (*RchiOBHmChr7g0241861*) in S7 of Cluster 119 may participate in flavonol biosynthesis. Furthermore, Cluster 119 was identified as an angiosperm-wide cluster, with nodes that were closely connected (Figure S3).

### 3.5. RcMYB114a May Affect the Formation of the Flower Color and Fragrance

**Figure 6:**
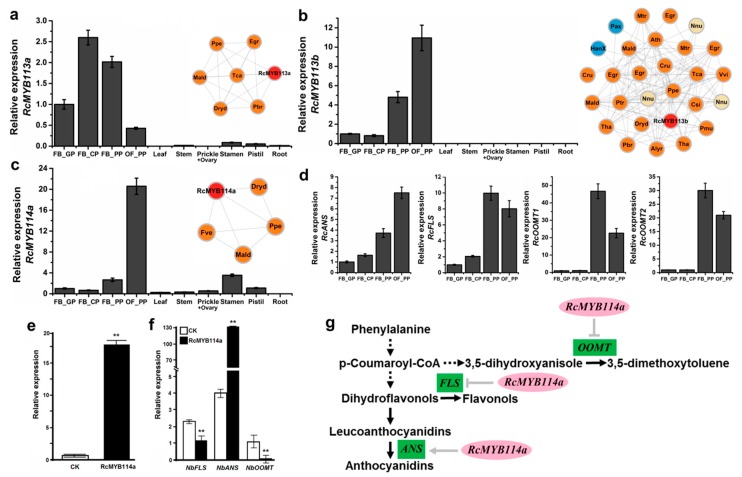
Gene expression and synteny associations of *R2R3-MYB* genes in Subgroup 6: (**a**–**c**) analysis of *RcMYB113a*, *RcMYB113b*, and *RcMYB114a* expression levels in four petal developmental stages and in other tissues based on a qRT-PCR assay. The following four typical petal developmental stages were examined: FB_GP (green petals in the flower bud), FB_CP (petals changing colors in the flower bud), FB_PP (pink petals in the flower bud), and OF_PP (pink petals on the open flower). Data are presented as the mean + standard deviation of three biological replicates. The primers for each gene are listed in Supplemental Table S16. The synteny network of *RcMYB113a* (Cluster 82), *RcMYB113b* (Cluster 2), and *RcMYB114a* (Cluster 54) are presented on the right. Node colors represent the following: rosids (orange), asterids (blue), *R. chinensis* (red), and other species (beige). Gene names and species abbreviations are provided in the nodes of rose and other species, respectively. (**d**) Analysis of *RcANS*, *RcFLS*, *RcOOMT1*, and *RcOOMT2* expression levels in four petal developmental stages. Data are presented as the mean + standard deviation of three biological replicates. (**e**) Transient overexpression of *RcMYB114a* in tobacco leaves. White bars represent the *RcMYB114a* expression level in control leaf samples treated with the empty vector, whereas black bars represent the *RcMYB114a* expression level. Data are presented as the mean + standard deviation of three biological replicates. ** *p* < 0.01 (Student’s *t*-test). (**f**) Expression of endogenous tobacco genes (*NbFLS*, *NbANS*, and *NbOOMT*) in the control and *RcMYB114a*-overexpressing leaf samples. Data are presented as the mean + standard deviation of three biological replicates. ** *p* < 0.01 (Student’s *t*-test). (**g**) Summary of the predicted regulatory functions of *RcMYB114a* in roses.

The five identified *RcMYB* genes (namely *RcMYB113a*, *RcMYB113b*, *RcMYB113c*, *RcMYB114a*, and *RcMYB114b*) in Subgroup 6 may be involved in anthocyanin biosynthesis. We completed a qRT-PCR assay to analyze the expression of these five genes in four petal developmental stages and in other tissues (Figure 6a–c and Supplemental Figure S4). The qRT-PCR results were consistent with the RNA-seq data (Figure 4c,d). These *RcMYB* genes were more highly expressed in petals than in the other examined tissues, especially *RcMYB113b*, which was specifically expressed in rose petals. Cluster 2, which includes *RcMYB113b*, was eudicot-specific (Figure 6b and Supplemental Table S4). Additionally, *RcMYB113a* and *RcMYB114a* belonged to the rosid-specific Clusters 82 and 54, respectively (Figure 6a,c). For further analyses, we selected the following genes encoding four key enzymes in the later steps of the phenylpropanoid pathway: *RcANS* (anthocyanidin synthase), *RcFLS* (flavonol synthase), *RcOOMT1* (orcinol O-methyltransferase 1), and *RcOOMT2* (orcinol O-methyltransferase 2). Both *RcOOMT1* and *RcOOMT2* were selected because of their roles in the biosynthesis of 3,5-dimethoxytoluene, which is one of the main compounds responsible for the *R. chinensis* flower fragrance [51,52]. A qRT-PCR assay was completed to analyze the expression of these genes during different petal developmental stages (Figure 6d). The *RcMYB114a* gene was co-expressed with *RcANS* from FB_GP to OF_PP, with expression trends that were the opposite to that of *RcFLS*, *RcOOMT1*, and *RcOOMT2* in FB_PP and OF_PP. Additionally, *RcMYB114a* was transiently expressed in tobacco leaves (Figure 6e), after which we examined whether the expression levels of the tobacco homologs of *RcANS*, *RcFLS*, *RcOOMT1*, and *RcOOMT2* were affected. Compared with the expression levels in the control leaf samples, which were injected with *A. tumefaciens* cells containing the empty vector, the *RcMYB114a*-overexpressing leaf samples had higher *NbANS* expression levels but lower *NbFLS* and *NbOOMT* expression levels (Figure 6f).

## 4. Discussion

The R2R3-MYB transcription factors have important regulatory functions, influencing almost all aspects of plant growth, development, and metabolism. The diversity of their functions is reflected by the size of the R2R3-MYB family. In the 35 species examined in our study, *R. chinensis* has 121 R2R3-MYBs, and only six species have fewer than 90 R2R3-MYBs (Supplemental Table S6). Synteny networks can clearly present the synteny among the *R2R3-MYB* genes in several plant genomes as well as the corresponding polyploidy events. Whole-genome duplication events can introduce new gene copies, leading to changes in evolutionary regulatory constraints, which are one of the main drivers of functional diversity within a gene family [53,54]. In the Rosaceae family, a WGD event occurred only in *Malus domestica* and *Pyrus × bretschneideri* (Figure 1b), which have 183 and 163 *R2R3-MYB* genes, respectively. These two species have considerably more *R2R3-MYB* genes than the other Rosaceae species analyzed in this study.

We applied a synnet analysis for the *R2R3-MYB* genes from 35 species, and the communities were labeled with the corresponding partial clustering ID numbers (Figure S1). The large clusters, such as Clusters 106, 107, and 108 (i.e., angiosperm-wide), had nodes that are likely to have important conserved functions. However, we focused more on the clusters with species-specific synteny to identify the special MYBs generated during the evolution of rose species, as they may be related to the unique traits of rose plants. Additionally, the seven rose MYB genes that exhibited synteny only with strawberry genes will need to be more thoroughly investigated (Figure 2h). We detected a tandem distribution of rose *R2R3-MYB* genes on Chr6 and the seven MYB genes corresponded to the nodes of Cluster 108 (Figure 3a,b). Cluster 108 indicated that the tandem distribution of *R2R3-MYB* genes is conserved among Rosaceae species. The effect of this phenomenon on the function of R2R3-MYB transcription factors in Rosaceae remains unknown, because the functions of these MYBs are mostly uncharacterized.

The phylogenetic tree with *RcMYB* and *AtMYB* genes provided considerable information regarding *RcMYB* functions. We were most interested in the transcriptional regulation of *RcMYB* genes in the phenylpropanoid pathway. A previous study summarized the R2R3-MYB-related regulation of the phenylpropanoid pathway in *Arabidopsis*, with a proposed model mainly comprising MYB genes from Subgroups 4–7 [2]. We tried to combine diverse information from clusters, spatiotemporal gene expression analyses, and multiple sequence alignments to investigate the R2R3-MYBs of the phenylpropanoid pathway in *R. chinensis*. Regarding S4, we speculated that *RcMYB308d* is conserved among angiosperms, based on the synnet results (Figure 5a). However, Cluster 59, which includes *RcMYB6c,* might have evolved relatively independently in monocots and typical eudicots (rosids and asterids), with the monocot cluster expanding during evolution (Figure 5b). The amino acid residues encoded by the genes in Clusters 112 and 59 also revealed differences, although the genes all belong to the same subgroup. The phylogenetic relationships and synnet revealed in this study may be useful for screening MYB transcription factors with important functions. In S5, *AtMYB123*, which is an ortholog of *RcTT2*, helps regulate proanthocyanidin synthesis [55]. Both *AtMYB123* and *RcTT2* belong to Cluster 108, which also contains the tandemly distributed *RcMYB* genes. The closest subgroup of S5 was S28, a rose-specific subgroup with unknown functions. Many of the *RcMYB* genes in Cluster 108 and S28 exhibit spatiotemporal expression trends that are similar to that of *RcTT2*. These *RcMYB* genes may need to be prioritized in future studies on the transcriptional regulation of proanthocyanidin synthesis in roses.

In plants, MYB transcription factors are important for regulating anthocyanin synthesis [56]. In petunias, the MYB transcription factors are affected by growth and development as well as environmental conditions, and they regulate the complex floral and vegetative tissue pigmentation patterns [57]. In red-skinned pear, *PyMYB114* is reportedly involved in anthocyanin biosynthesis [58]. Previous studies have also confirmed that MYB genes have important functions in many secondary metabolic processes [59,60]. In the current study, we determined that the four *RcMYB* genes in Subgroup 6 are highly expressed in rose petals at stages FB_PP and OF_PP (Figure 6b,c and Supplemental Figure S4), which coincide with the stages in which anthocyanins accumulate (mainly cynidin-3,5-diglucoside in ‘Old Blush’ petals) [49]. Furthermore, *OOMT* regulates the biosynthesis of the major compound responsible for the fragrance of rose flowers, 3,5-dimethoxytoluene, which is derived from the phenylpropanoid pathway [51,61]. Our data did not indicate that *ANS*, *FLS*, and *OOMT* transcription is directly regulated by *RcMYB114a*. However, our results provided some evidence of the complexity of the MYB functions in roses. The potential regulatory functions of *RcMYB114a* in the phenylpropanoid pathway are summarized in Figure 6g. These functions will be experimentally verified in future studies.

Analyses of shared synteny are critical for determining the orthology of genomic regions in various species. Moreover, a highly conserved synteny may reflect the functional relationships among genes [62,63]. The results of the current study indicate that plant *R2R3-MYB* genes have distinct origins, and the synteny of most of them is conserved among angiosperms. Independent evolutionary patterns of some clusters (e.g., tandem distribution and ancestral diversification) may have contributed to sequence diversification and neofunctionalizations. The structural and functional plasticity due to the distinct evolution of *R2R3-MYB* genes may have contributed to the rich ornamental traits of rose plants. The data presented herein may form the foundation of future investigations on the evolution and functions of homologs from multiple species. Our findings may also be relevant for identifying rose genetic resources with useful functions.

## Supplementary Materials

The following are available online at https://www.mdpi.com/2073-4425/10/10/823/s1. Figure S1: Network of all detected syntenic relationships among *R2R3-MYB* genes from 35 species. The clusters are distinguished based on color. Partial clustering ID numbers are indicated for the corresponding clusters. Figure S2: *Rosa chinensis* tissues that underwent an RNA-seq analysis. (**a**) The leaf (Le) samples from left to right are compound leaf, the adaxial side of a leaflet, and the abaxial side of a leaflet. (**b**) Pistil and ovary (PO) and stamen (Sta). (**c**) Stem (Ste) and prickle (Pri). (**d**) Root (Ro). (**e**) Pearson correlation analysis of the 12 samples. Figure S3: Synteny network of the *R2R3-MYB* genes in Cluster 119. The node colors indicate the species are monocots (green), rosids (pink), asterids (blue), or belong to other categories (beige). Two rose MYBs are indicated in red. Figure S4: Analysis of *RcMYB113c* and *RcMYB114b* expression in four petal samples and other tissues (namely root, stem, leaf, prickle, stamen, pistil, and ovary) of *R. chinensis* ‘Old Blush’. The internal control for the qRT-PCR assay is *RcActin*. Data are presented as the mean + standard deviation of three biological replicates. Table S1: Details regarding the 35 analyzed plant genomes. Table S2: Total node information of R2R3-MYBs in 35 species. Table S3: Total edge information of R2R3-MYBs in 35 species. Table S4: The nodelist of 119 characterized clusters (k = 3). Table S5: The edgelist of 119 characterized clusters (*k* = 3). Table S6: Number of syntenic genes and syntenic clusters of R2R3-MYBs in 35 species. Table S7: Evolutionary categories of syntenic *R2R3-MYB* genes in angiosperms. Table S8: Seven edges between *R. chinensis* and *F. vesca* (*k* < 3). Table S9: Physical positions of the 121 *R2R3-MYB* genes in the seven chromosome scaffolds of *R. chinensis.* Table S10: R2R3-MYB sequences used to construct a phylogenetic tree. Table S11: Information regarding the subgroup classification of R2R3-MYBs in *R. chinensis* and *A. thaliana.* Table S12: Positive selection of R2R3-MYBs in *R. chinensis* and *A. thaliana.* Table S13: FPKM values of *R. chinensis* R2R3-MYBs in four petal developmental stages. Table S14: Analysis of the read quality for 12 samples. Table S15: Number of RNA-seq reads sequenced and mapped on the *R. chinensis* genome. Table S16: FPKM values of R2R3-MYBs in six *R. chinensis* tissues. Table S17: Gene-specific primer sequences used in this study.

## References

[1] Riechmann J.L., Heard J., Martin G., Reuber L., Jiang C.-Z., Keddie J., Adam L., Pineda O., Ratcliffe O., Samaha R. (2000). *Arabidopsis* transcription factors: Genome-wide comparative analysis among eukaryotes. Science.

[2] Dubos C., Stracke R., Grotewold E., Weisshaar B., Martin C., Lepiniec L. (2010). MYB transcription factors in *Arabidopsis*. Trends Plant Sci..

[3] Mmadi M., Dossa K., Wang L., Zhou R., Wang Y., Cisse N., Sy M., Zhang X. (2017). Functional characterization of the versatile MYB gene family uncovered their important roles in plant development and responses to drought and waterlogging in sesame. Genes.

[4] Rogers L.A., Campbell M.M. (2004). The genetic control of lignin deposition during plant growth and development. New Phytol..

[5] Du H., Zhang L., Liu L., Tang X.F., Yang W.J., Wu Y.M., Huang Y.B., Tang Y.X. (2009). Biochemical and molecular characterization of plant MYB transcription factor family. Biochemistry (Moscow).

[6] Feller A., Machemer K., Braun E.L., Grotewold E. (2011). Evolutionary and comparative analysis of MYB and bHLH plant transcription factors. Plant J..

[7] Jin H., Martin C. (1999). Multifunctionality and diversity within the plant MYB-gene family. Plant Mol. Biol..

[8] Urao T., Yamaguchi-Shinozaki K., Urao S., Shinozaki K. (1993). An *Arabidopsis* myb homolog is induced by dehydration stress and its gene product binds to the conserved MYB recognition sequence. Plant Cell.

[9] Abe H., Yamaguchi-Shinozaki K., Urao T., Iwasaki T., Hosokawa D., Shinozaki K. (1997). Role of *Arabidopsis* MYC and MYB homologs in drought-and abscisic acid-regulated gene expression. Plant Cell.

[10] Gocal G.F., Sheldon C.C., Gubler F., Moritz T., Bagnall D.J., MacMillan C.P., Li S.F., Parish R.W., Dennis E.S., Weigel D. (2001). GAMYB-like genes, flowering, and gibberellin signaling in *Arabidopsis*. Plant Physiol..

[11] Jin H., Cominelli E., Bailey P., Parr A., Mehrtens F., Jones J., Tonelli C., Weisshaar B., Martin C. (2000). Transcriptional repression by AtMYB4 controls production of UV-protecting sunscreens in *Arabidopsis*. EMBO J..

[12] Urao T., Noji M.A., Yamaguchi-Shinozaki K., Shinozaki K. (1996). A transcriptional activation domain of ATMYB2, a drought-inducible *Arabidopsis* Myb-related protein. Plant J..

[13] Liu J., Osbourn A., Ma P. (2015). *MYB* transcription factors as regulators of phenylpropanoid metabolism in plants. Mol. Plant.

[14] Paz-Ares J., Ghosal D., Wienand U., Peterson P., Saedler H. (1987). The regulatory c1 locus of *Zea mays* encodes a protein with homology to myb proto-oncogene products and with structural similarities to transcriptional activators. EMBO J..

[15] Wilkins O., Nahal H., Foong J., Provart N.J., Campbell M.M. (2009). Expansion and diversification of the *Populus* R2R3-MYB family of transcription factors. Plant Physiol..

[16] Yanhui C., Xiaoyuan Y., Kun H., Meihua L., Jigang L., Zhaofeng G., Zhiqiang L., Yunfei Z., Xiaoxiao W., Xiaoming Q. (2006). The MYB transcription factor superfamily of *Arabidopsis*: Expression analysis and phylogenetic comparison with the rice MYB family. Plant Mol. Biol..

[17] Du H., Feng B.-R., Yang S.-S., Huang Y.-B., Tang Y.-X. (2012). The R2R3-MYB transcription factor gene family in maize. PLoS ONE.

[18] Du H., Yang S.-S., Liang Z., Feng B.-R., Liu L., Huang Y.-B., Tang Y.-X. (2012). Genome-wide analysis of the MYB transcription factor superfamily in soybean. BMC Plant Biol..

[19] Cao Z.-H., Zhang S.-Z., Wang R.-K., Zhang R.-F., Hao Y.-J. (2013). Genome wide analysis of the apple MYB transcription factor family allows the identification of *MdoMYB121* gene confering abiotic stress tolerance in plants. PLoS ONE.

[20] Soler M., Camargo E.L.O., Carocha V., Cassan-Wang H., San Clemente H., Savelli B., Hefer C.A., Paiva J.A.P., Myburg A.A., Grima-Pettenati J. (2015). The *Eucalyptus grandis* R2R3-MYB transcription factor family: Evidence for woody growth-related evolution and function. New Phytol..

[21] Jiang C., Gu X., Peterson T. (2004). Identification of conserved gene structures and carboxy-terminal motifs in the Myb gene family of *Arabidopsis* and *Oryza sativa* L. ssp. indica. Genome Biol..

[22] Liu C., Wang X., Xu Y., Deng X., Xu Q. (2014). Genome-wide analysis of the R2R3-MYB transcription factor gene family in sweet orange (*Citrus sinensis*). Mol. Biol. Rep..

[23] Lee T.H., Tang H., Wang X., Paterson A.H. (2012). PGDD: A database of gene and genome duplication in plants. Nucleic Acids Res..

[24] Jiao Y., Li J., Tang H., Paterson A.H. (2014). Integrated syntenic and phylogenomic analyses reveal an ancient genome duplication in monocots. Plant Cell.

[25] Thomas C. (2000). Modern Roses XI: The World Encyclopedia of Roses.

[26] Raymond O., Gouzy J., Just J., Badouin H., Verdenaud M., Lemainque A., Vergne P., Moja S., Choisne N., Pont C. (2018). The Rosa genome provides new insights into the domestication of modern roses. Nat. Genet..

[27] Tanaka Y., Sasaki N., Ohmiya A. (2008). Biosynthesis of plant pigments: Anthocyanins, betalains and carotenoids. Plant J..

[28] Falcone Ferreyra M.L., Rius S., Casati P. (2012). Flavonoids: Biosynthesis, biological functions, and biotechnological applications. Front. Plant Sci..

[29] Zvi M.M.B., Shklarman E., Masci T., Kalev H., Debener T., Shafir S., Ovadis M., Vainstein A. (2012). *PAP1* transcription factor enhances production of phenylpropanoid and terpenoid scent compounds in rose flowers. New Phytol..

[30] Bateman A., Coin L., Durbin R., Finn R.D., Hollich V., Griffiths-Jones S., Khanna A., Marshall M., Moxon S., Sonnhammer E.L. (2004). The Pfam protein families database. Nucleic Acids Res..

[31] Finn R.D., Clements J., Eddy S.R. (2011). HMMER web server: Interactive sequence similarity searching. Nucleic Acids Res..

[32] Zhao T., Schranz M.E. (2017). Network approaches for plant phylogenomic synteny analysis. Curr. Opin. Plant Biol..

[33] Wang Y., Tang H., DeBarry J.D., Tan X., Li J., Wang X., Lee T.-H., Jin H., Marler B., Guo H. (2012). MCScanX: A toolkit for detection and evolutionary analysis of gene synteny and collinearity. Nucleic Acids Res..

[34] Shannon P., Markiel A., Ozier O., Baliga N.S., Wang J.T., Ramage D., Amin N., Schwikowski B., Ideker T. (2003). Cytoscape: A software environment for integrated models of biomolecular interaction networks. Genome Res..

[35] Bastian M., Heymann S., Jacomy M. (2009). Gephi: An open source software for exploring and manipulating networks. Icwsm.

[36] Palla G., Derényi I., Farkas I., Vicsek T. (2005). Uncovering the overlapping community structure of complex networks in nature and society. Nature.

[37] Fortunato S. (2010). Community detection in graphs. Phys. Rep..

[38] Dixon P. (2003). VEGAN, a package of R functions for community ecology. J. Veg. Sci..

[39] Voorrips R. (2002). MapChart: Software for the graphical presentation of linkage maps and QTLs. J. Hered..

[40] Larkin M.A., Blackshields G., Brown N., Chenna R., McGettigan P.A., McWilliam H., Valentin F., Wallace I.M., Wilm A., Lopez R. (2007). Clustal W and Clustal X version 2.0. Bioinformatics.

[41] Tamura K., Stecher G., Peterson D., Filipski A., Kumar S. (2013). MEGA6: Molecular evolutionary genetics analysis version 6.0. Mol. Biol. Evol..

[42] Letunic I., Bork P. (2016). Interactive tree of life (iTOL) v3: An online tool for the display and annotation of phylogenetic and other trees. Nucleic Acids Res..

[43] Librado P., Rozas J. (2009). DnaSP v5: A software for comprehensive analysis of DNA polymorphism data. Bioinformatics.

[44] Pertea M., Pertea G.M., Antonescu C.M., Chang T.C., Mendell J.T., Salzberg S.L. (2015). StringTie enables improved reconstruction of a transcriptome from RNA-seq reads. Nat. Biotechnol..

[45] Kim D., Langmead B., Salzberg S.L. (2015). HISAT: A fast spliced aligner with low memory requirements. Nat. Methods.

[46] Anders S., Pyl P.T., Huber W. (2015). HTSeq- a Python framework to work with high-throughput sequencing data. Bioinformatics.

[47] Trapnell C., Williams B.A., Pertea G., Mortazavi A., Kwan G., Van Baren M.J., Salzberg S.L., Wold B.J., Pachter L. (2010). Transcript assembly and quantification by RNA-Seq reveals unannotated transcripts and isoform switching during cell differentiation. Nat. Biotechnol..

[48] Anders S., Huber W. (2012). Differential expression of RNA-Seq data at the gene level–the DESeq package. Heidelb. Ger. Eur. Mol. Biol. Lab. (EMBL).

[49] Han Y., Wan H., Cheng T., Wang J., Yang W., Pan H., Zhang Q. (2017). Comparative RNA-seq analysis of transcriptome dynamics during petal development in *Rosa chinensis*. Sci. Rep..

[50] Zhao T., Holmer R., de Bruijn S., Angenent G.C., van den Burg H.A., Schranz M.E. (2017). Phylogenomic synteny network analysis of MADS-box transcription factor genes reveals lineage-specific transpositions, ancient tandem duplications, and deep positional conservation. Plant Cell.

[51] Scalliet G., Journot N., Jullien F., Baudino S., Magnard J.-L., Channelière S., Vergne P., Dumas C., Bendahmane M., Cock J.M. (2002). Biosynthesis of the major scent components 3, 5-dimethoxytoluene and 1, 3, 5-trimethoxybenzene by novel rose O-methyltransferases. FEBS Lett..

[52] Lavid N., Wang J., Shalit M., Guterman I., Bar E., Beuerle T., Menda N., Shafir S., Zamir D., Adam Z. (2002). O-methyltransferases involved in the biosynthesis of volatile phenolic derivatives in rose petals. Plant Physiol..

[53] Conant G.C., Wolfe K.H. (2008). Turning a hobby into a job: How duplicated genes find new functions. Nat. Rev. Genet..

[54] Flagel L.E., Wendel J.F. (2009). Gene duplication and evolutionary novelty in plants. New Phytol..

[55] Liu Y., Shi Z., Maximova S.N., Payne M.J., Guiltinan M.J. (2015). Tc-MYBPA is an *Arabidopsis* TT2-like transcription factor and functions in the regulation of proanthocyanidin synthesis in Theobroma cacao. BMC Plant Biol..

[56] Quattrocchio F., Wing J.F., Leppen H.T., Mol J.N., Koes R.E. (1993). Regulatory genes controlling anthocyanin pigmentation are functionally conserved among plant species and have distinct sets of target genes. Plant Cell.

[57] Albert N.W., Lewis D.H., Zhang H., Schwinn K.E., Jameson P.E., Davies K.M. (2011). Members of an R2R3-MYB transcription factor family in *Petunia* are developmentally and environmentally regulated to control complex floral and vegetative pigmentation patterning. Plant J..

[58] Yao G., Ming M., Allan A.C., Gu C., Li L., Wu X., Wang R., Chang Y., Qi K., Zhang S. (2017). Map-based cloning of the pear gene *MYB 114* identifies an interaction with other transcription factors to coordinately regulate fruit anthocyanin biosynthesis. Plant J..

[59] Wei X., Liu K., Zhang Y., Feng Q., Wang L., Zhao Y., Li D., Zhao Q., Zhu X., Zhu X. (2015). Genetic discovery for oil production and quality in sesame. Nat. Commun..

[60] Bhatia C., Pandey A., Gaddam S.R., Hoecker U., Trivedi P.K. (2018). Low temperature-enhanced flavonol synthesis requires light-associated regulatory components in *Arabidopsis thaliana*. Plant Cell Physiol..

[61] Scalliet G., Piola F., Douady C.J., Réty S., Raymond O., Baudino S., Bordji K., Bendahmane M., Dumas C., Cock J.M. (2008). Scent evolution in Chinese roses. Proc. Natl. Acad. Sci. USA.

[62] Dewey C.N. (2011). Positional orthology: Putting genomic evolutionary relationships into context. Brief. Bioinform..

[63] Altenhoff A.M., Boeckmann B., Capella-Gutierrez S., Dalquen D.A., DeLuca T., Forslund K., Huerta-Cepas J., Linard B., Pereira C., Pryszcz L.P. (2016). Standardized benchmarking in the quest for orthologs. Nat. Methods.

