# Supplementary material for: Dissecting the Genome-Wide Evolution and Function of R2R3-MYB Transcription Factor Family in Rosa chinensis
Source: Genes (Basel). 2019 Oct 18;10(10):823. doi: 10.3390/genes10100823 (PMC6826493; doi:10.3390/genes10100823)
Supplement: Supplementary file 1 [file genes-10-00823-s001.zip › Supplementary Files/Supplementary Information.docx]

**Table S1.** Details regarding the 35 analyzed plant genomes.

| **Species_Name** | **Species_Name** | **Abbreviation** | **Clade1** | **Clade2** | **Clade3** | **Version** | **Reference** |
| --- | --- | --- | --- | --- | --- | --- | --- |
| *Xerophyta viscosa* | *Xerophyta viscosa* | Xvis | Monocots | Pandanales | Velloziaceae | ASM207613v1 | Costa et al., 2017 |
| *Oropetium thomaeum* | *Oropetium thomaeum* | oth | Monocots | Poales | Poaceae | Oropetium_genomic_20141112 | VanBuren et al., 2015 |
| *Triticum turgidum* | Wheat | Trdc | Monocots | Poales | Poaceae | Version 1.0 | Avni et al., 2017 |
| *Oryza sativa* | Rice | osa | Monocots | Poales | Poaceae | Version 7.0 | International Rice Genome Sequencing, 2005 |
| *Zea mays* | Maize | Zmay | Monocots | Poales | Poaceae | Version 6a | Schnable et al., 2009 |
| *Musa acuminata* | Banana | mac | Monocots | Zingiberales | Musaceae | July_2012 | D'Hont et al., 2012 |
| *Apostasia shenzhenica* | Orchid | Ashe | Monocots | Asparagales | Orchidaceae | ASM278626v1 | Zhang et al., 2017 |
| *Phalaenopsis equestris* | Orchid | peq | Monocots | Asparagales | Orchidaceae | Version 5.0 | Cai et al., 2015 |
| *Pyrus* × *bretschneideri* | Pear | pbr | Rosid I/Fabids | Rosales | Rosaceae | Version 1.0 | Wu et al., 2013 |
| *Malus domestica* | Apple | Mald | Rosid I/Fabids | Rosales | Rosaceae | MalDomGD1.0 | Daccord et al., 2017 |
| *Rosa chinensis* | Rose | Rchi | Rosid I/Fabids | Rosales | Rosaceae | RchiOBHm-V2 | Raymond et al., 2018 |
| *Fragaria vesca* | Strawberry | fve | Rosid I/Fabids | Rosales | Rosaceae | Version 1.1 | Shulaev et al., 2011 |
| *Dryas drummondii* | *Dryas drummondii* | Dryd | Rosid I/Fabids | Rosales | Rosaceae | ASM325486v1 | Griesmann et al., 2018 |
| *Prunus persica* | Peach | ppe | Rosid I/Fabids | Rosales | Rosaceae | Prunus_persica_NCBIv2 | Verde et al., 2013 |
| *Prunus mume* | Mei | pmu | Rosid I/Fabids | Rosales | Rosaceae | Version 1.0 | Zhang et al., 2012 |
| *Citrullus lanatus* | Watermelon | cla | Rosid I/Fabids | Cucurbitales | Cucurbitaceae | CiLa_1.0 | Guo et al., 2013 |
| *Glycine max* | Soybean | gma | Rosid I/Fabids | Fabales | Fabaceae | Glycine_max_v2.1 | Schmutz et al., 2010 |
| *Medicago truncatula* | Barrel medic | mtr | Rosid I/Fabids | Fabales | Fabaceae | MedtrA17_4.0 | Young et al., 2011 |
| *Populus trichocarpa* | Western poplar | ptr | Rosid I/Fabids | Malpighiales | Salicaceae | Pop_tri_v3 | Tuskan et al., 2006 |
| *Capsella rubella* | Capsella | cru | Rosid II/Malvids | Brassicales | Brassicaceae | Caprub1_0 | Slotte et al., 2013 |
| *Arabidopsis thaliana* | Arabidopsis | ath | Rosid II/Malvids | Brassicales | Brassicaceae | TAIR10.1 | Swarbreck et al., 2007 |
| *Arabidopsis lyrata* | Lyrate rockcress | Alyr | Rosid II/Malvids | Brassicales | Brassicaceae | Version 1.0 | Hu et al., 2011 |
| *Tarenaya hassleriana* | Tarenaya | tha | Rosid II/Malvids | Brassicales | Cleomaceae | ASM46358v1 | Cheng et al., 2013 |
| *Theobroma cacao* | Cacao | tca | Rosid II/Malvids | Malvales | Malvaceae | Criollo_cocoa_genome_V2 | Argout et al., 2011 |
| *Citrus sinensis* | Sweet orange | csi | Rosid II/Malvids | Sapindales | Rutaceae | Csi_valencia_1.0 | Xu et al., 2013 |
| *Eucalyptus grandis* | Eucalyptus | egr | Rosid II/Malvids | Myrtales | Myrtaceae | Egrandis1_0 | Myburg et al., 2014 |
| *Vitis vinifera* | Grape vine | vvi | Rosids | Vitales | Vitaceae | Genoscope | Jaillon et al., 2007 |
| *Solanum tuberosum* | Potato | stu | Asterid I/Lamiids | Solanales | Solanaceae | SolTub_3.0 | Potato Genome Sequencing et al., 2011 |
| *Solanum lycopersicum* | Tomato | sly | Asterid I/Lamiids | Solanales | Solanaceae | SL3.0 | Tomato Genome, 2012 |
| *Petunia axillaris* | Petunia | pax | Asterid I/Lamiids | Solanales | Solanaceae | Version 1.0 | Bombarely et al., 2016 |
| *Chrysanthemum nankingense* | Chrysanthemum | Cind | Asterid II/Campanulids | Asterales | Asteraceae | Version 1.0 | Song et al., 2018 |
| *Helianthus annuus* | Sunflower | HanX | Asterid II/Campanulids | Asterales | Asteraceae | HanXRQr1.0 | Badouin et al., 2017 |
| *Beta vulgaris* | Sugar beet | bvu | Eudicots | Caryophyllales | Asteraceae | RefBeet-1.2.2 | Dohm et al., 2014 |
| *Nelumbo nucifera* | Chinese Lotus | nnu | Eudicots | Nymphaeales | Nelumbonaceae | Chinese Lotus 1.1 | Ming et al., 2013 |
| *Amborella trichopoda* | Amborella | atr | Angiosperms | Amborellales | Amborellaceae | AMTR1.0 | Albert et al., 2013 |

**Table S8.** The list of 7 edges between *R. chinensis* and *F. vesca.* (k < 3).

| **Locus_1** | **Locus_2** |
| --- | --- |
| Rchi112178144 (RchiOBHmChr7g0229011) | fve_25060 |
| Rchi112178735 (RchiOBHmChr7g0219951) | fve_29254 |
| Rchi112180306 (RchiOBHmChr7g0235271) | fve_26513 |
| Rchi112190178 (RchiOBHmChr2g0142471) | fve_23113 |
| Rchi112192427 (RchiOBHmChr1g0335311) | fve_23544 |
| Rchi112199927 (RchiOBHmChr4g0389841) | fve_32606 |
| Rchi112203525 (RchiOBHmChr5g0032281) | fve_27215 |

**Table S11.** Information regarding the subgroup classification of R2R3-MYBs in *R. chinensis* and *A. thaliana.*

| **Subgroup** | **Cluster_ID of RcMYBs** | ***R.chinensis*_ID** | **Genome_ID** | ***A.thaliana*_ID** | **Genome_ID** | **Function prediction** | **References** |
| --- | --- | --- | --- | --- | --- | --- | --- |
| **S1** | Cluster107 | RcMYB30 | RchiOBHmChr3g0495721 | AtMYB31 | AT1G74650 |  | Froidure S et al., 2010;  Vailleau F et al., 2002;  Li L et al., 2009;  Zheng Y et al., 2012;  Rusconi F et al., 2013;  Cominelli E et al., 2005;  Wang F F et al., 2010;  Li L et al., 2009;  Raffaele S et al., 2008;  Seo P J et al., 2009;  Seo P J et al., 2010 |
|  | Cluster107 | RcMYB30a | RchiOBHmChr2g0168581 | AtMYB30 | AT3G28910 | 1. Abiotic stress response / HR response 2. Hypocotyl elongation 3. Brassinosteroid pathway, related to the regulation of brassinosteroid-induced gene expression  4. Related to the biosynthesis of very-long-chain fatty acids involved in hypersensitive cell death |  |
|  | Cluster107 | RcMYB306b | RchiOBHmChr7g0181361 | AtMYB60 | AT1G08810 | 1. Biotic stress response / Drought, ABA-mediated (stomatal closure) 2. Regulating light-induced stomatal aperture |  |
|  |  |  |  | AtMYB94 | AT3G47600 |  |  |
|  |  |  |  | AtMYB96 | AT5G62470 | 1. Abiotic and biotic stress response / Drought and pathogens ABA- and JA-mediated 2. ABA/auxin cross-talker, mediating ABA signaling during drought stress and involved in promoting pathogen resistance |  |
| **S2** | Cluster106 | RcMYB14 | RchiOBHmChr4g0392981 | AtMYB13 | AT1G06180 | Abiotic stress response / Drought, light and wounding, ABA-mediated (shoot morphogenesis) | Cominelli E et al., 2005;  Seo P J et al., 2009;  Kirik V et al., 1998 |
|  |  |  |  | AtMYB14 | AT2G31180 | 1. Response to auxin/cold/ethylene/freezing/jasmonic acid/salicylic acid/salt stress 2. Involved in cold tolerance/an important element in the signaling pathway of cold tolerance |  |
|  |  |  |  | AtMYB15 | AT3G23250 | 1. Cold response 2. Drought and salt, ABA-mediated |  |
| **S3** | Cluster10 | RcMYB63 | RchiOBHmChr5g0025771 | AtMYB10 | AT3G12820 | The most highly induced transcription factors within 24 hours of exposure to iron deficiency | Zhou J et al., 2009;  Segarra G et al., 2009;  Van der Ent S et al., 2008 |
|  |  |  |  | AtMYB58 | AT1G16490 | Phenylpropanoide pathway / Lignin biosynthesis (fibers and vessels) |  |
|  |  |  |  | AtMYB63 | AT1G79180 | Phenylpropanoide pathway / Lignin biosynthesis (fibers and vessels) |  |
|  |  |  |  | AtMYB72 | AT1G56160 | 1. Biotic stress response / Pathogens (induced systemic resistance) 2. Required for induced systemic resistance (ISR) in Arabidopsis roots |  |
| **S4** | Cluster59 | RcMYB6c | RchiOBHmChr7g0228621 | AtMYB3 | AT1G22640 | Phenylpropanoide pathway | Dubos C et al., 2008; Jin H et al., 2000; Preston J et al., 2004 |
|  | Cluster112 | RcMYB308d | RchiOBHmChr6g0252211 | AtMYB4 | AT4G38620 | 1. Phenylpropanoide pathway / Sinapate ester biosynthesis 2. Regulate UV stress and wound responses in Arabidopsis |  |
|  |  |  |  | AtMYB6 | AT4G09460 | Can bind to the binding site of mammal c-MYB and that of maize P gene product |  |
|  |  |  |  | AtMYB7 | AT2G16720 | 1. Phenylpropanoide pathway/Light-regulated.  2. Can bind to the binding site of mammal c-MYB and that of maize P gene product |  |
|  |  |  |  | AtMYB8 | AT1G35515 |  |  |
|  |  |  |  | AtMYB32 | AT4G34990 | Phenylpropanoide pathway |  |
| **S5** | Cluster108 | RcTT2 | RchiOBHmChr2g0094481 | AtMYB123 | AT5G35550 | Phenylpropanoide pathway / Proanthocyanindins biosynthesis | Zhou M L et al., 2013;  Nesi N et al., 2001 |
| **S6** | Cluster82 | RcMYB113a | RchiOBHmChr3g0448721 | AtMYB75 | AT1G56650 | 1. Phenylpropanoide pathway / Anthocyanin biosynthesis 2. Activators involved in anthocyanins biosynthesis in Arabidopsis 3. Activation of PAP1 (AtMYB75) accounts for the bright-purple phenotype | Appelhagen I et al., 2011;  Borevitz J O et al., 2000;  Gonzalez A et al., 2008 |
|  | Cluster2 | RcMYB113b | RchiOBHmChr3g0492711 | AtMYB90 | AT1G66390 | 1. Phenylpropanoide pathway / Anthocyanin biosynthesis 2. Activators involved in anthocyanins biosynthesis in Arabidopsis |  |
|  | Rchi+fve | RcMYB113c | RchiOBHmChr7g0235271 | AtMYB113 | AT1G66370 | 1. Phenylpropanoide pathway / Anthocyanin biosynthesis 2. Activators involved in anthocyanins biosynthesis in Arabidopsis |  |
|  | Cluster54 | RcMYB114a | RchiOBHmChr2g0116041 | AtMYB114 | AT1G66380 | Phenylpropanoide pathway / Anthocyanin biosynthesis |  |
|  |  | RcMYB114b | RchiOBHmChr2g0116071 |  |  |  |  |
| **S7** | Cluster119 | RcMYB30c | RchiOBHmChr7g0241861 | AtMYB111 | AT5G49330 | Phenylpropanoide pathway / Flavonol biosynthesis | Stracke R et al., 2007;  Mehrtens F et al., 2005;  Pandey A et al., 2015 |
|  |  |  |  | AtMYB11 | AT3G62610 | 1. Phenylpropanoide pathway / Flavonol biosynthesis 2. Flavonol-specific regulators which positively modulate expression of biosynthetic genes involved in flavonol biosynthesis 3. AtMYB11 had a weaker effect on enhancing flavonol biosynthesis as compared to AtMYB12 and AtMYB111 in activating flavonoid biosynthesis. |  |
|  |  |  |  | AtMYB12 | AT2G47460 | Phenylpropanoide pathway / Flavonol biosynthesis |  |
| **S9** | Cluster107 | RcMYB106 | RchiOBHmChr2g0167441 | AtMYB16 | AT5G15310 | Cell fate / Conical epidermal cell outgrowth | Baumann K et al., 2007;  Zhang Y et al., 2009;  Jakoby M J et al., 2008;  Li S F et al., 2009;  Gonzalez A et al., 2009 |
|  | Cluster107 | RcMYB16 | RchiOBHmChr4g0439371 | AtMYB106 | AT3G01140 | Cell fate / Trichome branching |  |
|  | Cluster64 | RcMYB41 | RchiOBHmChr1g0354061 | AtMYB17 | AT3G61250 |  |  |
|  | Cluster107 | RcMYB106L | RchiOBHmChr7g0181821 |  |  |  |  |
|  | Cluster107 | RcMYB41Lb | RchiOBHmChr7g0181801 |  |  |  |  |
| **S10** | Cluster51 | RcMYB39 | RchiOBHmChr7g0210281 | AtMYB9 | AT5G16770 |  |  |
|  |  |  |  | AtMYB107 | AT3G02940 |  |  |
|  |  |  |  | AtMYB39 | AT4G17785 |  |  |
| **S11** | Cluster106 | RcMYB102 | RchiOBHmChr5g0006031 | AtMYB74 | AT4G05100 |  | Lippold F et al., 2009;  De Vos M et al., 2006 |
|  | Cluster106 | RcMYB41La | RchiOBHmChr2g0161391 | AtMYB102 | AT4G21440 |  |  |
|  |  |  |  | AtMYB41 | AT4G28110 | Abiotic stress response / Osmotic, ABA-mediated |  |
|  |  |  |  | AtMYB49 | AT5G54230 |  |  |
| **S12** |  |  |  | AtMYB29 | AT5G07690 | Glucosinolate biosynthesis / Aliphatic pool | Gigolashvili T et al., 2007;  Gigolashvili T et al., 2008;  Celenza J L et al., 2005;  Seo M S et al., 2016 |
|  |  |  |  | AtMYB34 | AT5G60890 | Glucosinolate biosynthesis / Indolic pool Activates tryptophan gene expression in Arabidopsis |  |
|  |  |  |  | AtMYB51 | AT1G18570 | Glucosinolate biosynthesis / Indolic pool |  |
|  |  |  |  | AtMYB76 | AT5G07700 | Glucosinolate biosynthesis / Aliphatic pool |  |
|  |  |  |  | AtMYB122 | AT1G74080 | Glucosinolate biosynthesis / Indolic pool |  |
|  |  |  |  | AtMYB28 | AT5G61420 | Regulates aliphatic GSL biosynthesis |  |
| **S13** | Cluster15 | RcMYB86b | RchiOBHmChr7g0192781 | AtMYB86 | AT5G26660 |  | Penfield S et al., 2001;  Newman L J et al., 2004;  Liang Y K et al., 2005 |
|  | Cluster102 | RcMYB61a | RchiOBHmChr3g0458721 | AtMYB50 | AT1G57560 |  |  |
|  |  |  |  | AtMYB61 | AT1G09540 | 1. Mucillage deposition and extrusion 2 .Phenylpropanoide pathway / Lignin biosynthesis 3. Stomatal closure 4. MYB61 is required for mucilage deposition and extrusion in the Arabidopsis seed coat; AtMYB61 miss expressed in det3 mutant that can explain the ectopic signification and dark-photomorphogenic phenotype of the det3 mutant. |  |
|  |  |  |  | AtMYB55 | AT4G01680 |  |  |
| **S14** | Cluster111 | RcRAX3a | RchiOBHmChr2g0109131 | AtMYB68 | AT5G65790 | Root elongation (abiotic stress response: temperature) | Keller T et al., 2006;  Müller D et al., 2006;  Hong S H et al., 2008;  Feng C et al., 2004 |
|  | Cluster111 | RcRAX3b | RchiOBHmChr6g0310231 | AtMYB84 | AT3G49690 | Axillary meristem regulation / Lateral organ formation (shoot branching, GA-mediated) |  |
|  | Cluster113 | RcMYB36 | RchiOBHmChr4g0403351 | AtMYB36 | AT5G57620 |  |  |
|  | Cluster98 | RcRAX2 | RchiOBHmChr3g0470381 | AtMYB37 | AT5G23000 | Axillary meristem regulation / Lateral organ formation (shoot branching, GA-mediated) |  |
|  | Cluster98 | RcRAX2L | RchiOBHmChr5g0057501 | AtMYB38 | AT2G36890 | 1. Axillary meristem regulation / Lateral organ formation (shoot branching, GA-mediated) 2. Hypoctyle elongation, blue light-mediated (CRY1 signalling) |  |
|  | Cluster111 | RcRAX1 | RchiOBHmChr2g0092411 | AtMYB87 | AT4G37780 |  |  |
| **S15** | Cluster105 | RcWERg | RchiOBHmChr7g0193591 | AtMYB0 | AT3G27920 | Plays a central role in trichome initiation. | Oppenheimer D G et al., 1991;  Kirik V et al., 2005;  Kang Y H et al., 2009;  Lee M M et al., 1999 |
|  |  |  |  | AtMYB23 | AT5G40330 | 1. Cell fate / Root hair patterning 2. Trichome initiation and branching |  |
|  |  |  |  | AtMYB66 | AT5G14750 | Cell fate / Root hair patterning |  |
| **S16** | Cluster115 | RcLAF1 | RchiOBHmChr1g0377201 | AtMYB18 | AT4G25560 | Hypoctyle elongation, Far red light-mediated (phyA signalling) | Yang S W et al., 2009 |
|  |  |  |  | AtMYB19 | AT5G52260 |  |  |
|  |  |  |  | AtMYB45 | AT3G48920 |  |  |
| **S18** | Cluster41 | RcMYB101La | RchiOBHmChr5g0040211 | AtMYB97 | AT4G26930 |  | Millar A A et al., 2005;  Reyes J L et al., 2007;  Zhong R et al., 2007 |
|  |  | RcMYB101Lb |  | AtMYB120 | AT5G55020 |  |  |
|  | Cluster41 | RcMYB101 | RchiOBHmChr2g0159111 | AtMYB101 | AT2G32460 | Abiotic stress response / ABA sensitivity |  |
|  | Cluster68 | RcMYB33 | RchiOBHmChr1g0329681 | AtMYB33 | AT5G06100 | 1. Stamen development / Anther development (tapetum) 2. Abiotic stress response / ABA sensitivity |  |
|  |  |  |  | AtMYB65 | AT3G11440 | Stamen development / Anther development (tapetum) |  |
|  |  |  |  | AtMYB81 | AT2G26960 |  |  |
|  |  |  |  | AtMYB104 | AT2G26950 |  |  |
| **S19** | Cluster104 | RcMYB305a | RchiOBHmChr2g0172331 | AtMYB21 | AT3G27810 | Stamen development / Filament length, GA- and JA-mediated | Mandaokar A et al., 2006 |
|  |  |  |  | AtMYB24 | AT5G40350 | Stamen development / Filament length, dehiscence process, GA- and JA mediated |  |
|  |  |  |  | AtMYB57 | AT3G01530 | Stamen development / Filament length, GA- and JA-mediated |  |
| **S20** | Cluster118 | RcMYB24b | RchiOBHmChr4g0434891 | AtMYB62 | AT1G68320 | Abiotic stress response / Phosphate starvation, GA-mediated | Mandaokar A. 2009;  Abe H et al., 2003;  Devaiah B N et al., 2009 |
|  |  | RcMYB62a | RchiOBHmChr6g0267581 | AtMYB116 | AT1G25340 |  |  |
|  | Cluster118 | RcMYB62b | RchiOBHmChr7g0185561 | AtMYB2 | AT2G47190 | Abiotic stress response / Drought and salt, ABA-mediated |  |
|  |  |  |  | AtMYB78 | AT5G49620 |  |  |
|  |  |  |  | AtMYB108 | AT3G06490 | 1. Stamen development / Pollen maturation, dehiscence process, GA- and JA-mediated 2. Abiotic and biotic / Dehydration, wounding and pathogens, JA mediated |  |
|  |  |  |  | AtMYB112 | AT1G48000 |  |  |
| **S21** | Rchi+fve | RcCSA | RchiOBHmChr7g0219951 | AtMYB52 | AT1G17950 | Cell wall thickening (fibers) | Zhong R et al., 2008;  Lee D K et al., 2009 |
|  | Cluster52 | RcMYB52 | RchiOBHmChr7g0177631 | AtMYB54 | AT1G73410 | Cell wall thickening (fibers) |  |
|  | Cluster100 | RcMYB54a | RchiOBHmChr4g0439571 | AtMYB56 | AT5G17800 |  |  |
|  |  | RcMYB54b | RchiOBHmChr7g0219961 | AtMYB69 | AT4G33450 | Cell wall thickening (fibers) |  |
|  | Cluster50 | RcMYB73La | RchiOBHmChr6g0277601 | AtMYB89 | AT5G39700 |  |  |
|  |  |  |  | AtMYB105 | AT1G69560 | Axillary meristem regulation / Lateral organ formation |  |
|  |  |  |  | AtMYB110 | AT3G29020 |  |  |
|  |  |  |  | AtMYB117 | AT1G26780 | Axillary meristem regulation / Lateral organ formation |  |
| **S22** | Cluster101 | RcMYB44a | RchiOBHmChr6g0307001 | AtMYB44 | AT5G67300 | Abiotic stress response / Drought, salt and cold, ABA-mediated (stomatal closure) | Jung C et al., 2008;  Shin R et al., 2007 |
|  | Cluster101 | RcMYB44b | RchiOBHmChr6g0308731 | AtMYB70 | AT2G23290 | Abiotic stress response / Drought, Light, Wounding |  |
|  |  | RcMYB73 | RchiOBHmChr2g0106671 | AtMYB73 | AT4G37260 | Abiotic stress response / Drought, Light, Wounding |  |
|  | Cluster109 | RcMYB73Lb | RchiOBHmChr1g0370581 | AtMYB77 | AT3G50060 | 1. Growth regulation, auxin-mediated 2. Abiotic stress response / Drought, Light, Wounding |  |
| **S23** | Cluster109 | RcMYB1 | RchiOBHmChr3g0464251 | AtMYB1 | AT3G09230 |  |  |
|  | Cluster109 | RcMYB1L | RchiOBHmChr1g0369061 | AtMYB25 | AT2G39880 |  |  |
|  |  |  |  | AtMYB109 | AT3G55730 |  |  |
| **S24** | Cluster97 | RcMYB92 | RchiOBHmChr2g0113131 | AtMYB93 | AT1G34670 |  |  |
|  | Cluster97 | RcMYB93a | RchiOBHmChr7g0238891 | AtMYB53 | AT5G65230 |  |  |
|  |  | RcMYB93b | RchiOBHmChr7g0238951 | AtMYB92 | AT5G10280 |  |  |
| **S25** | Cluster11 | RcMYB98 | RchiOBHmChr7g0193411 | AtMYB98 | AT4G18770 | Cell fate / Synergid cell differentiation | Shinozaki K et al., 1992;  Wang X et al., 2009;  Zhang Y et al., 2009;  Lai L B et al., 2005;  Xie Z et al., 2010;  Kasahara R D et al., 2005 |
|  |  | RcMYB119 | RchiOBHmChr7g0211831 | AtMYB64 | AT5G11050 |  |  |
|  | Cluster99 | RcMYB64 | RchiOBHmChr5g0068891 | AtMYB119 | AT5G58850 |  |  |
|  |  |  |  | AtMYB22 | AT5G40430 |  |  |
|  |  |  |  | AtMYB100 | AT2G25230 |  |  |
|  |  |  |  | AtMYB115 | AT5G40360 | Embryogenesis |  |
|  |  |  |  | AtMYB118 | AT3G27785 | Embryogenesis / Seed maturation |  |
| **S26** | Cluster107 | RcMYB35 | RchiOBHmChr2g0169671 | AtMYB35 | AT3G28470 | Stamen development / Anther development (tapetum) |  |
|  | Cluster49 | RcMYB80 | RchiOBHmChr5g0075031 | AtMYB80 | AT5G56110 | Stamen development / Anther (tapetum) and pollen (exine)development |  |
|  |  | RcMYB35L | RchiOBHmChr7g0180151 |  |  |  |  |
| **S27** | Cluster114 | RcODO1a | RchiOBHmChr1g0374861 | AtMYB42 | AT4G12350 |  |  |
|  | Cluster114 | RcODO1b | RchiOBHmChr7g0202031 | AtMYB85 | AT4G22680 | Lignin deposition / Cell wall thickening (fiber cells) |  |
|  | Cluster79 | RcMYB20 | RchiOBHmChr3g0491061 | AtMYB43 | AT5G16600 |  |  |
|  |  |  |  | AtMYB20 | AT1G66230 |  |  |
|  |  |  |  | AtMYB99 | AT5G62320 | Stamen development / Anther development (tapetum) |  |
| **S28** |  | RcWERa | RchiOBHmChr2g0142501 |  |  |  |  |
|  | Rchi+fve | RcWERb | RchiOBHmChr4g0389841 |  |  |  |  |
|  |  | RcWERc | RchiOBHmChr5g0039751 |  |  |  |  |
|  | Cluster108 | RcWERd | RchiOBHmChr6g0311731 |  |  |  |  |
|  | Rchi+fve | RcMYB114c | RchiOBHmChr2g0142471 |  |  |  |  |
|  | Rchi+fve | RcMYB3a | RchiOBHmChr6g0311841 |  |  |  |  |
|  |  | RcGL1b | RchiOBHmChr2g0146961 |  |  |  |  |
| **S29** | Cluster45 | RcMYB21 | RchiOBHmChr2g0143681 | AtMYB26 | AT3G13890 | Stamen development / Anther development (endothecium) |  |
|  | Cluster89 | RcMYB86a | RchiOBHmChr5g0023221 | AtMYB67 | AT3G12720 |  |  |
|  | Cluster89 | RcMYB61b | RchiOBHmChr5g0021591 |  |  |  |  |
| **S30** | Cluster117 | RcMYB46 | RchiOBHmChr5g0051961 | AtMYB46 | AT5G12870 | Cell wall thickening (fibers and vessels) |  |
|  | Cluster117 | RcMYB46L | RchiOBHmChr1g0315931 | AtMYB83 | AT3G08500 |  |  |
| **S31** | Cluster104 | RcMYB24a | RchiOBHmChr2g0164981 | AtMYB121 | AT3G30210 |  |  |
|  | Cluster19 | RcMYB340 | RchiOBHmChr4g0396001 | AtMYB71 | AT3G24310 |  |  |
|  |  |  |  | AtMYB79 | AT4G13480 |  |  |
| **S32** | Cluster66 | RcSRM1 | RchiOBHmChr4g0446981 | AtDHL5 | AT5G08520 |  |  |
|  | Rchi+fve | RcDIVa | RchiOBHmChr1g0335311 | AtDHL3 | AT5G04760 |  |  |
|  | Cluster103 | RcDIVc | RchiOBHmChr5g0068581 | AtHL2 |  |  |  |
|  | Cluster103 | RcDIVb | RchiOBHmChr1g0369341 | AtDHL6 | AT1G49010 |  |  |
|  |  |  |  | AtHL1 | AT5G23650 |  |  |
|  |  |  |  | AtTF2 | AT3G11280 |  |  |
|  |  |  |  | AtDHL4 | AT5G05790 |  |  |
|  |  |  |  | AtDHL1 | AT2G38090 |  |  |
|  |  |  |  | AtDHL2 |  |  |  |
| none subgroup | Cluster51 | RcMYB53 | RchiOBHmChr1g0384441 |  |  |  |  |
| none subgroup | Cluster44 | RcMYB5 |  | AtMYB5 | AT3G13540 | 1. Mucilage biosynthesis 2. Phenylpropanoide pathway / Proanthocyanindins biosynthesis 3. Light-induced. Expressed in trichome, stipule epidermal cells of the developing leaves and in immature seeds | Li S F et al., 2009;  Gonzalez A et al., 2009 |
| none subgroup |  |  |  | AtMYB95 |  |  |  |
| none subgroup |  |  |  | AtMYB47 |  |  |  |
| none subgroup | Cluster38 | RcMYB315 | RchiOBHmChr2g0174081 | AtMYB40 | AT5G14340 |  |  |
| none subgroup | Cluster105 | RcMYB8f | RchiOBHmChr7g0186441 |  |  |  |  |
| none subgroup | Cluster105 | RcMYB308a(30869/RhMYBPA1) | RchiOBHmChr1g0360311 |  |  |  |  |
| none subgroup | Cluster94 | RcGL1a | RchiOBHmChr2g0087781 | AtMYB82 | AT5G52600 |  |  |
| none subgroup | Cluster108 | RcMYB8e | RchiOBHmChr6g0311701 |  |  |  |  |
| none subgroup | Cluster95 | RcWERh | RchiOBHmChr7g0203911 |  |  |  |  |
| none subgroup | Cluster108 | RcWERf | RchiOBHmChr6g0311771 |  |  |  |  |
| none subgroup | Cluster73 | RcMYB8c | RchiOBHmChr6g0304811 |  |  |  |  |
| none subgroup |  | RcMYB114d | RchiOBHmChr2g0152151 |  |  |  |  |
| none subgroup | Cluster108 | RcMYB6a | RchiOBHmChr2g0145221 |  |  |  |  |
| none subgroup | Cluster90 | RcARC1 | RchiOBHmChr2g0094491 |  |  |  |  |
| none subgroup | Cluster108 | RcMYB8d | RchiOBHmChr6g0311681 |  |  |  |  |
| none subgroup | Cluster85 | RcMYB8a | RchiOBHmChr2g0094451 |  |  |  |  |
| none subgroup | Cluster108 | RcMYB308f | RchiOBHmChr2g0094411 |  |  |  |  |
| none subgroup | Rchi+fve | RcWERi | RchiOBHmChr7g0229011 |  |  |  |  |
| none subgroup | Cluster108 | RcMYB30b | RchiOBHmChr6g0311711 |  |  |  |  |
| none subgroup | Cluster108 | RcWERe | RchiOBHmChr6g0311741 |  |  |  |  |
| none subgroup | Cluster108 | RcMYB308b | RchiOBHmChr2g0145191 |  |  |  |  |
| none subgroup |  | RcMYB305b | RchiOBHmChr7g0196711 | AtMYB103 |  |  |  |
| none subgroup | Cluster96 | RcMYB8g | RchiOBHmChr7g0196701 |  |  |  |  |
| none subgroup |  | RcMYB6b | RchiOBHmChr4g0441081 |  |  |  |  |
| none subgroup | Cluster93 | RcMYB308e | RchiOBHmChr7g0178681 |  |  |  |  |
| none subgroup |  | RcMYB3b | RchiOBHmChr7g0178691 |  |  |  |  |
| none subgroup | Cluster119 | RcMYBP | RchiOBHmChr4g0424331 |  |  |  |  |
| none subgroup | Cluster106 | RcMYB4b | RchiOBHmChr6g0288151 |  |  |  |  |
| none subgroup | Cluster106 | RcMYB8b | RchiOBHmChr5g0005721 |  |  |  |  |
| none subgroup | Cluster12 | RcMYB308c | RchiOBHmChr2g0161281 |  |  |  |  |
| none subgroup |  | RcMYB63L | RchiOBHmChr7g0234171 |  |  |  |  |
| none subgroup | Cluster106 | RcMYB4a | RchiOBHmChr2g0161271 |  |  |  |  |
| none subgroup | Cluster106 | RcMYB4c | RchiOBHmChr2g0161241 |  |  |  |  |
| none subgroup | Cluster71 | RcMYB27 | RchiOBHmChr3g0470621 | AtMYB27 |  |  |  |
| none subgroup |  |  |  | AtMYB48 |  |  |  |
| none subgroup |  |  |  | AtMYB59 |  | Root elongation (cell cycle regulation) | Mu R L et al., 2009 |
| none subgroup | Cluster84 | RcDUO1 | RchiOBHmChr1g0346571 | AtDUO1 |  |  |  |
| none subgroup | Cluster110 | RcAS1 | RchiOBHmChr3g0466491 | AtMYB91 |  | Axillary meristem regulation / Lateral organ separation (Leaves) | Byrne M E et al., 2000 |
| none subgroup | Cluster104 | RcRS2H | RchiOBHmChr2g0163191 |  |  |  |  |
| none subgroup | Cluster116 | RcMYB124 | RchiOBHmChr4g0440161 | AtMYB88 |  |  |  |
| none subgroup |  |  |  | AtMYB124 |  | Cell fate / Stomata cell differentiation | Lai L B et al., 2005;  Xie Z et al., 2010 |
| none subgroup |  | RcSMH4 |  |  |  |  |  |
| none subgroup |  |  |  | AtDHL7 | AT5G45420 |  |  |
| none subgroup | Rchi+fve | RcCDC5b | RchiOBHmChr5g0032281 |  |  |  |  |
| none subgroup | Cluster106 | RcCDC5a | RchiOBHmChr3g0449801 |  |  |  |  |

**Table S14.** Analysis of the read quality for 12 samples. Error (%) represent the error rate of sequenced bases. The error rate of all samples is not more than 0.02%.

| **Sample name** | **Raw reads** | **Clean reads** | **Clean bases** | **Error (%)** | **Q20 (%)** | **Q30 (%)** | **GC (%)** |
| --- | --- | --- | --- | --- | --- | --- | --- |
| Le_1 | 55723132 | 54773656 | 8.22G | 0.03 | 97.45 | 93.01 | 46.82 |
| Le_2 | 51893058 | 50798432 | 7.62G | 0.03 | 97.69 | 93.51 | 47.15 |
| Ste_1 | 57840718 | 56474700 | 8.47G | 0.03 | 97.57 | 93.22 | 46.75 |
| Ste_2 | 46726758 | 45297720 | 6.79G | 0.03 | 97.63 | 93.41 | 46.87 |
| Pri_1 | 53382190 | 52180504 | 7.83G | 0.03 | 97.75 | 93.63 | 46.99 |
| Pri_2 | 57070648 | 55525636 | 8.33G | 0.03 | 97.7 | 93.58 | 46.9 |
| Sta_1 | 53213330 | 52346290 | 7.85G | 0.03 | 97.48 | 93.06 | 46.36 |
| Sta_2 | 61610954 | 60223228 | 9.03G | 0.03 | 97.64 | 93.4 | 46.61 |
| PO_1 | 59345890 | 58024852 | 8.7G | 0.03 | 97.55 | 93.16 | 46.6 |
| PO_2 | 59586630 | 58536032 | 8.78G | 0.03 | 97.55 | 93.2 | 46.57 |
| Ro_1 | 56808682 | 55571306 | 8.34G | 0.03 | 97.68 | 93.55 | 47.88 |
| Ro_2 | 54661878 | 53865936 | 8.08G | 0.03 | 97.69 | 93.57 | 48.41 |

**Table S15.** Number of RNA-seq reads sequenced and mapped on the *R. chinensis* genome.

| **Sample_name** | **Le_1** | **Le_2** | **Ste_1** | **Ste_2** | **Pri_1** | **Pri_2** | **Sta_1** | **Sta_2** | **PO_1** | **PO_2** | **Ro_1** | **Ro_2** |
| --- | --- | --- | --- | --- | --- | --- | --- | --- | --- | --- | --- | --- |
| Total reads | 54773656 | 50798432 | 56474700 | 45297720 | 52180504 | 55525636 | 52346290 | 60223228 | 58024852 | 58536032 | 55571306 | 53865936 |
| Total mapped | 49704294 (90.74%) | 46599448 (91.73%) | 51237565 (90.73%) | 41055281 (90.63%) | 47697832 (91.41%) | 50647951 (91.22%) | 47466322 (90.68%) | 54879790 (91.13%) | 52450166 (90.39%) | 52712804 (90.05%) | 42459055 (76.4%) | 39802643 (73.89%) |
| Multiple mapped | 1254464 (2.29%) | 1116111 (2.2%) | 1246830 (2.21%) | 970995 (2.14%) | 1130537 (2.17%) | 1159148 (2.09%) | 1404006 (2.68%) | 1630013 (2.71%) | 1371688 (2.36%) | 1376656 (2.35%) | 1166651 (2.1%) | 1067671 (1.98%) |
| Uniquely mapped | 48449830 (88.45%) | 45483337 (89.54%) | 49990735 (88.52%) | 40084286 (88.49%) | 46567295 (89.24%) | 49488803 (89.13%) | 46062316 (88%) | 53249777 (88.42%) | 51078478 (88.03%) | 51336148 (87.7%) | 41292404 (74.31%) | 38734972 (71.91%) |

**Table S17.** Gene-specific primer sequences used in this study.

| **Gene_name** | **Gene_ID** | **Upper primers** | **Lower primers** |
| --- | --- | --- | --- |
| qPCR-*RcMYB113a* | RchiOBHmChr3g0448721 | GTGGCTAAACTACCTTCGGC | TCACATCATTACCAGTCCTTCC |
| qPCR-*RcMYB113b* | RchiOBHmChr3g0492711 | AGAGGTGGCTTTACGGAGG | CATCGTTTCCAGTTCGTCC |
| qPCR-*RcMYB113c* | RchiOBHmChr7g0235271 | AGGCAACAGGTGGTCATTG | GGTTTAGGTCTTAGTATGATGGGC |
| qPCR-*RcMYB114a* | RchiOBHmChr2g0116041 | AACAGCACCACCACAAACTC | GAAGTCGTCCTCTAAACCAAGAC |
| qPCR-*RcMYB114b* | RchiOBHmChr2g0116071 | TTTGGGAAACAGGTGGTCTT | ATGTCTCTCCTTCGCCGA |
| qPCR-*RcFLS* | RchiOBHmChr1g0327961 | ATGGGAGATGGAGCAGTGG | AGTAAGGTGAGGGCGGACA |
| qPCR-*RcANS* | RchiOBHmChr7g0199941 | TCTCACTTGGCTTGGGATTAG | TTATGTCGGTGTGGGCTTC |
| qPCR-*RcOOMT1* | RchiOBHmChr2g0128091 | CAATCCATCCAACCAAATCC | TGGGAAGCATCAGTAAGGGT |
| qPCR-*RcOOMT2* | RchiOBHmChr2g0119291 | TGGCCCCATGACTCTTTCTGA | AGAAGGAGCTGGGAAGCATCA |
| qPCR-*RcActin* | RchiOBHmChr3g0466761 | CAATGCTCCCGCTATGTATG | AGGTCAAGTCGCAGAATGG |
| qPCR-*NbFLS* | Niben101Scf02429g06007.1 | CAGGGAAGCAAATGAGGAATAC | CCAAGCCCAAGTGATAAACTCT |
| qPCR-*NbANS* | Niben101Scf00490g01007.1 | AAATCGACTCAGAGGACAAGG | CGCGATCAATTAGCTGACC |
| qPCR-*NbOOMT* | Niben101Scf03085g07013.1 | GCCAATGCCCTTTCAATC | CTTGGATTCTGGACTTGACG |
| qPCR-*NbActin* | Niben101Scf09773g01014.1 | CCTGATGGGCAAGTGATTAC | AGTTGTATGTGGTCTCGTGGA |
| OE-*RcMYB114a* | RchiOBHmChr2g0116041 | GGGGACAAGTTTGTACAAAAAAGCAGGCTTCATGGAGGGTTTCGGCGTG | GGGGACCACTTTGTACAAGAAAGCTGGGTTTTATGCGTAGAAGTTGTTGACTAGATCA |

**Supplementary Figures**


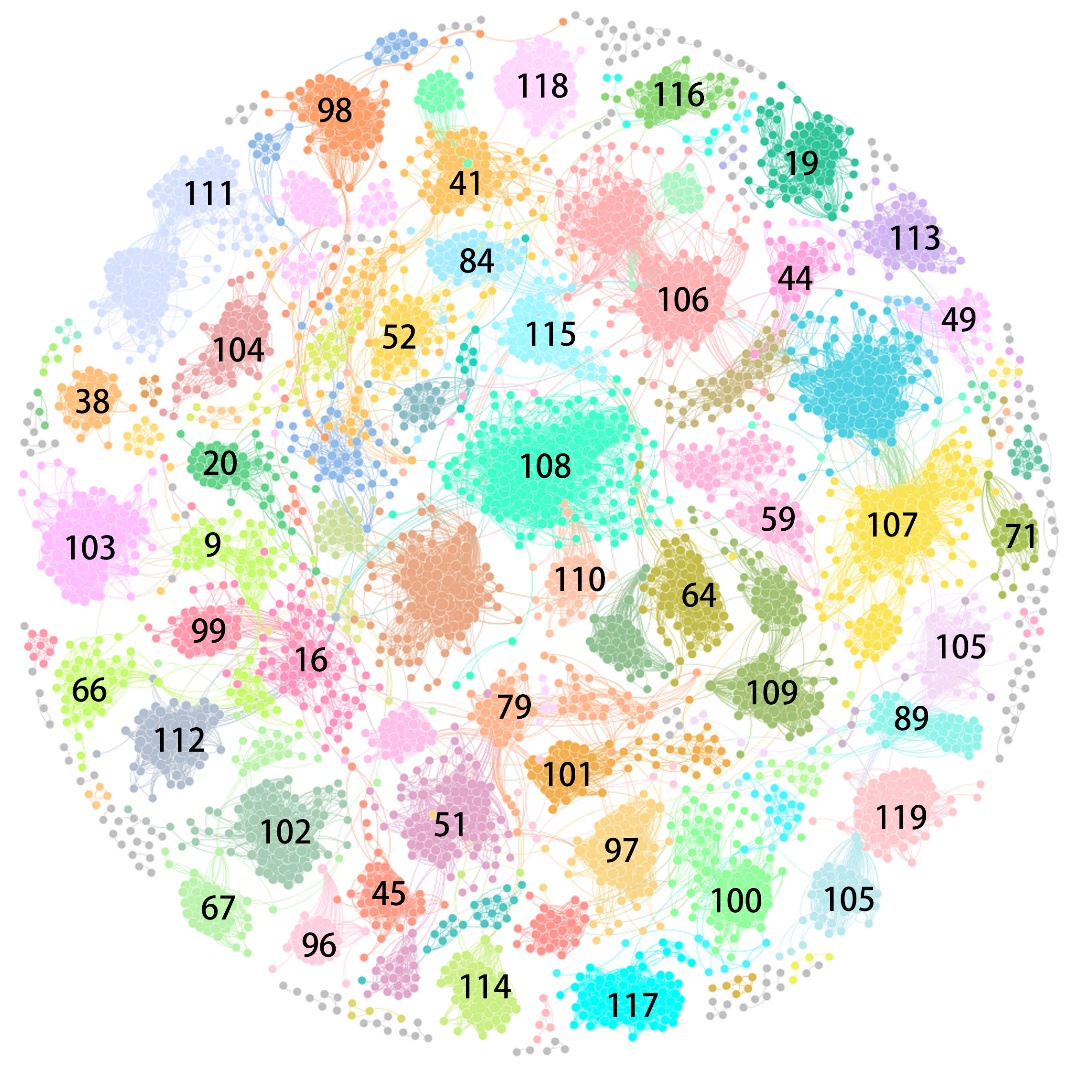


**Figure S1.** Network of all detected syntenic relationships among R2R3-MYB genes from 35 species. The clusters are distinguished based on color. Partial clustering ID numbers are indicated for the corresponding clusters.


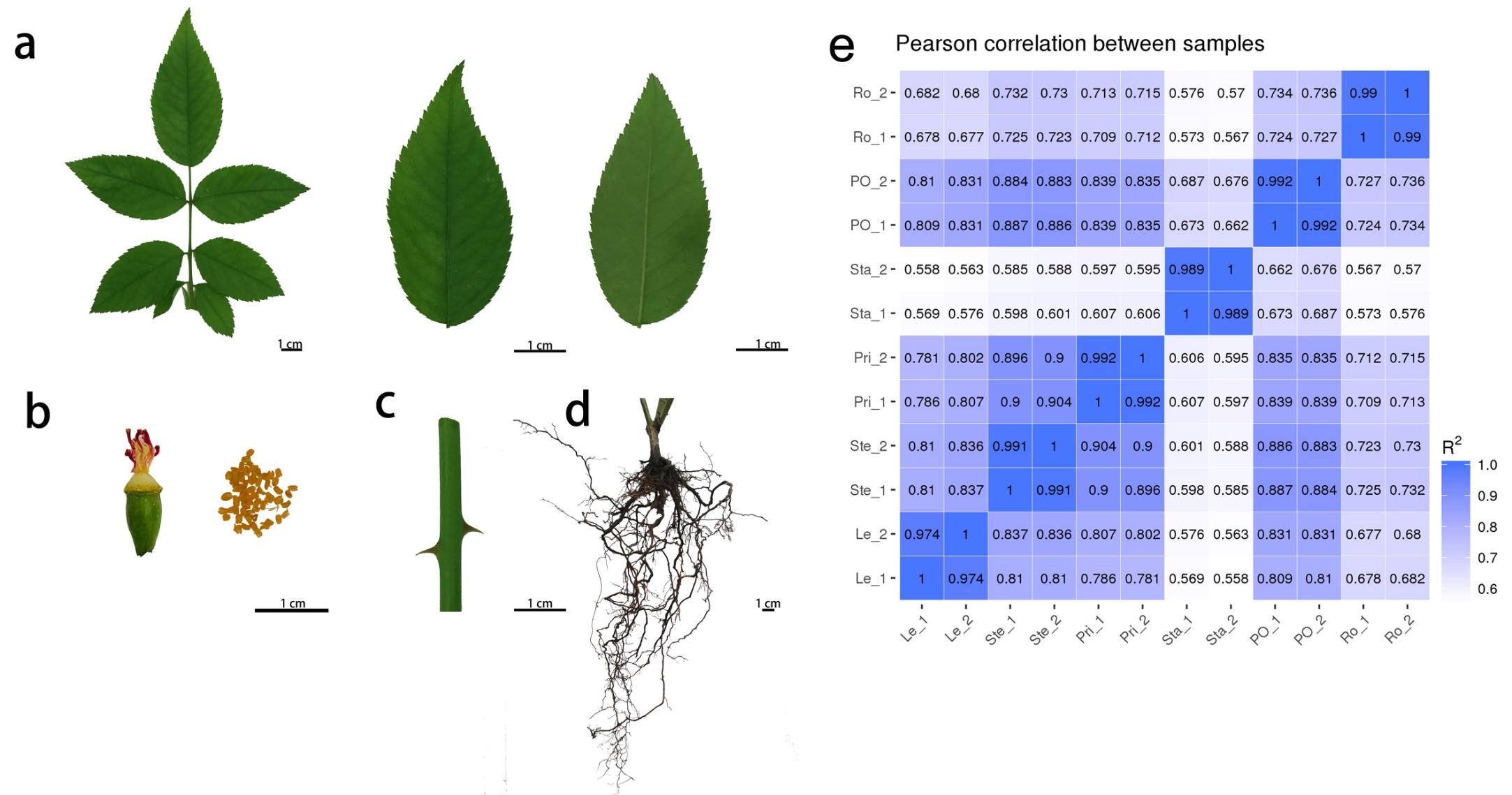


**Figure S2.** *Rosa chinensis* tissues that underwent an RNA-seq analysis. (a) The leaf (Le) samples from left to right are compound leaf, the adaxial side of a leaflet, and the abaxial side of a leaflet. (b) Pistil and ovary (PO) and stamen (Sta). (c) Stem (Ste) and prickle (Pri). (d) Root (Ro). (e) Pearson correlation analysis of the 12 samples.


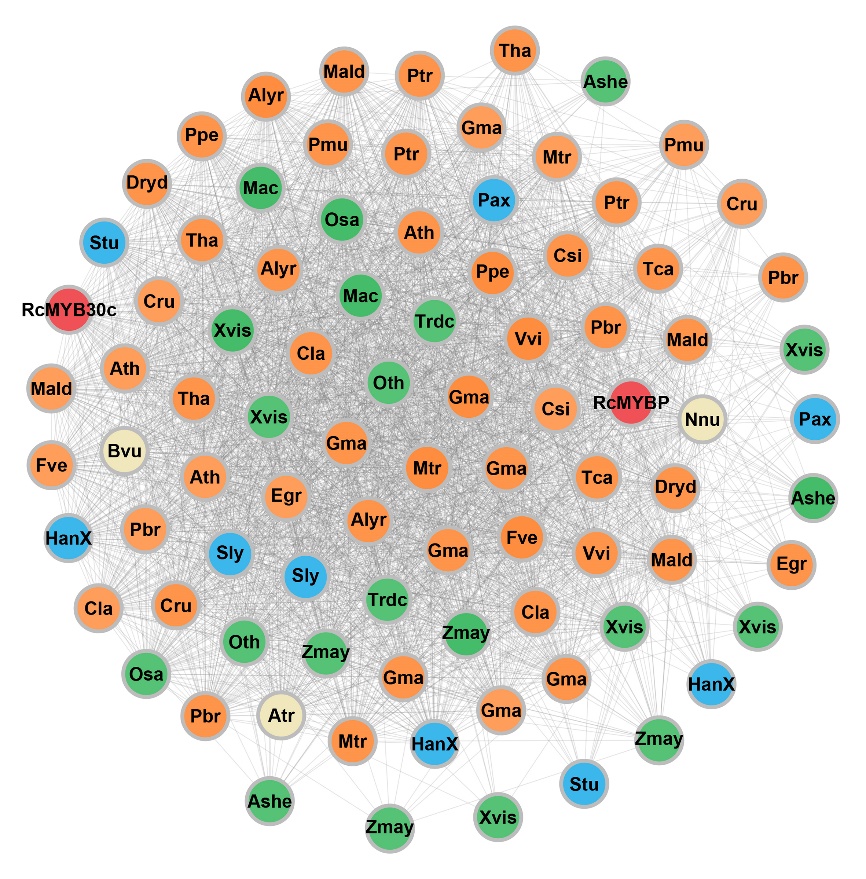


**Figure S3.** Synteny network of the R2R3-MYB genes in Cluster 119. The node colors indicate the species are monocots (green), rosids (pink), asterids (blue), or belong to other categories (beige). Two rose MYBs are indicated in red.


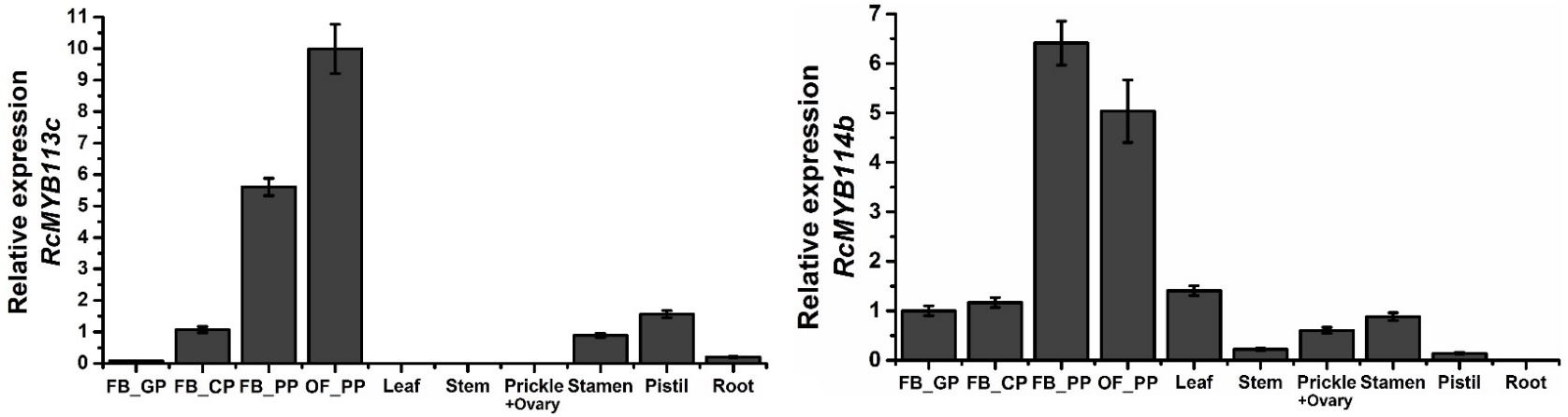


**Figure S4.** Analysis of *RcMYB113c* and *RcMYB114b* expression in four petal samples and other tissues (i.e., root, stem, leaf, prickle, stamen, pistil, and ovary) of *R. chinensis* ‘Old Blush’. The internal control for the qRT-PCR assay is *RcActin*. Data are presented as the mean + standard deviation of three biological replicates.

|  |  |
| --- | --- |
