# Supplementary figures and images for: Dissecting the Genome-Wide Evolution and Function of R2R3-MYB Transcription Factor Family in Rosa chinensis
Source: Genes (Basel). 2019 Oct 18;10(10):823. doi: 10.3390/genes10100823 (PMC6826493; doi:10.3390/genes10100823)

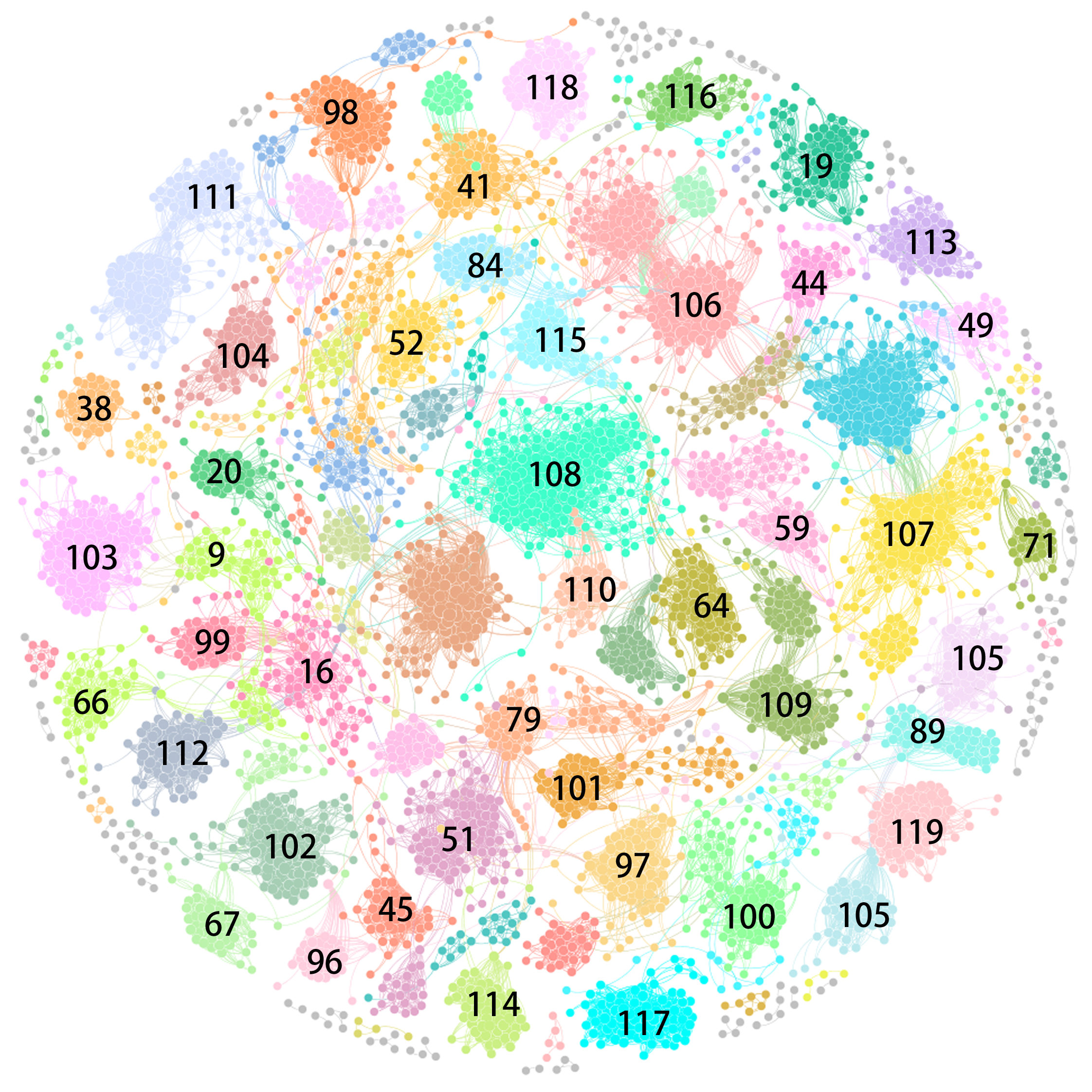

Supplement: Supplementary file 1 [file genes-10-00823-s001.zip › Supplementary Files/Figure S1.jpg]

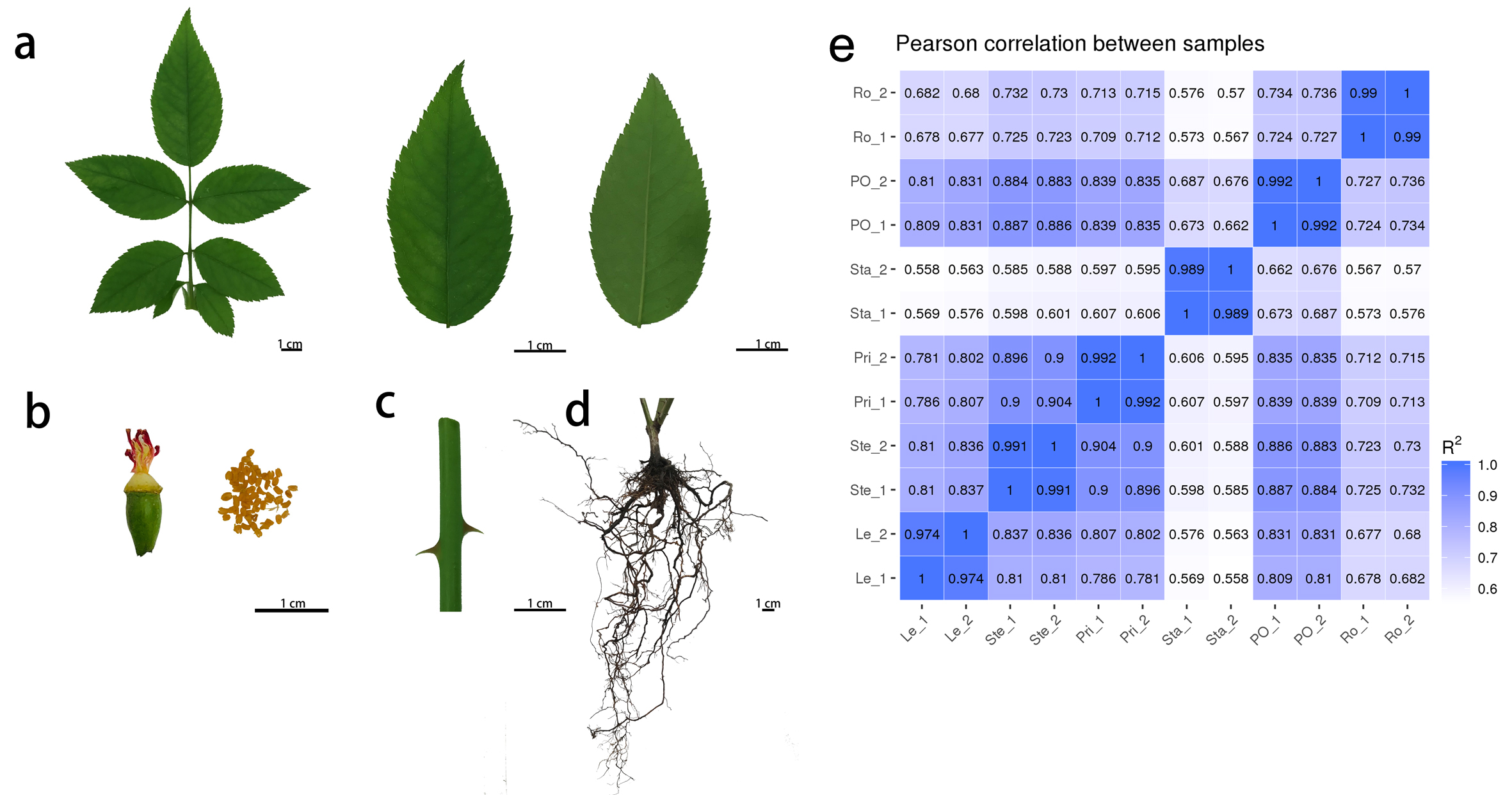

Supplement: Supplementary file 1 [file genes-10-00823-s001.zip › Supplementary Files/Figure S2.jpg]

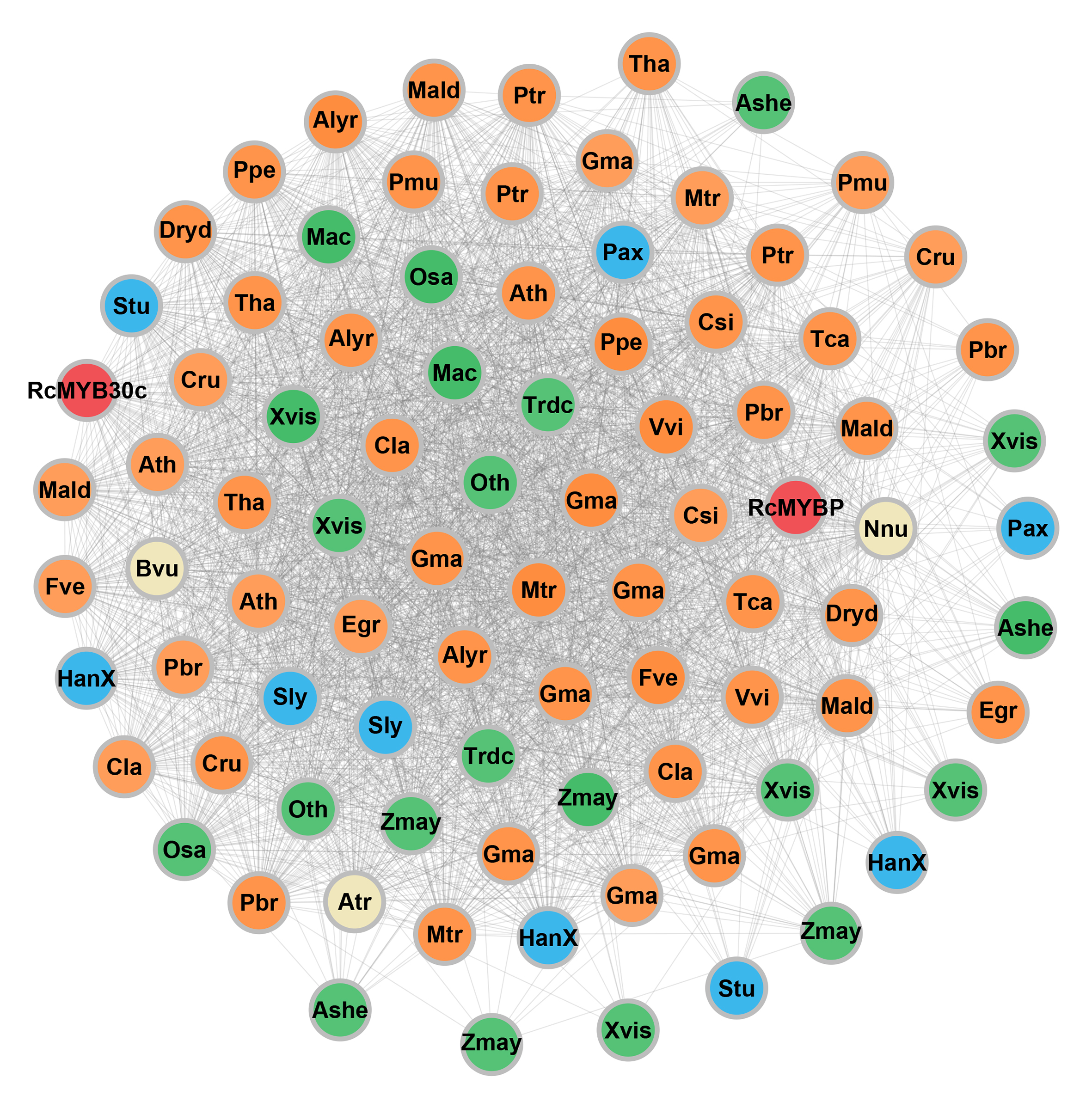

Supplement: Supplementary file 1 [file genes-10-00823-s001.zip › Supplementary Files/Figure S3.jpg]

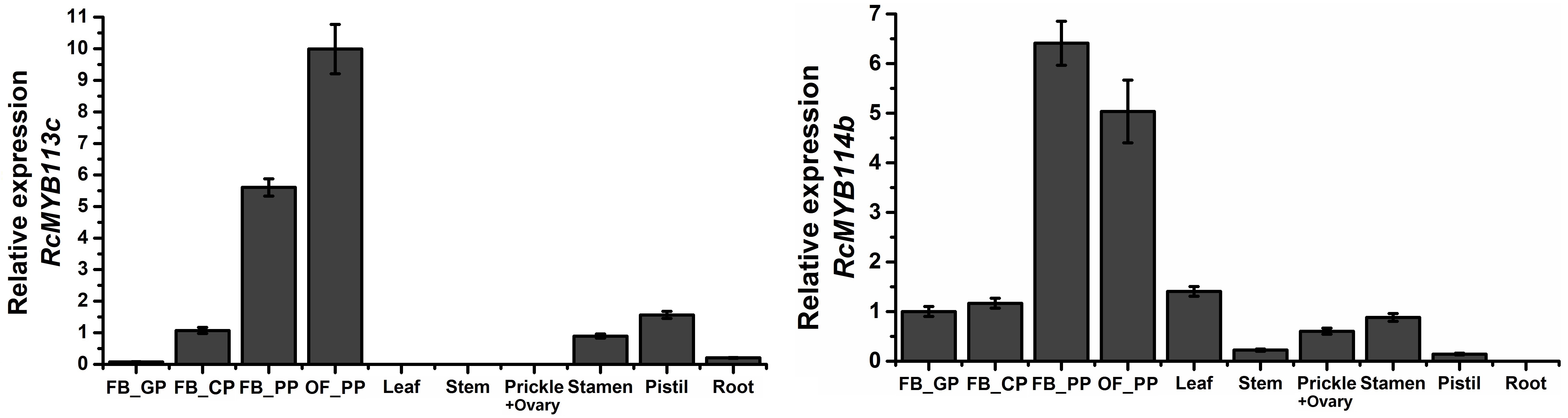

Supplement: Supplementary file 1 [file genes-10-00823-s001.zip › Supplementary Files/Figure S4.jpg]
